# Low protein-induced-FGF-21 signaling remodels adipose tissue on reduced markers of senescence during aging

**DOI:** 10.1007/s11357-025-01853-w

**Published:** 2025-09-29

**Authors:** Jose G. Godoy-Lugo, Khristina E. Young, Prerana Vaddi, Yvann Batamack, Jolaiya Aldridge, Sun Ok Fernandez-Kim, Diana C. Albarado, Susan J. Burke, Jacqueline M. Stephens, Christopher D. Morrison, Cristal M. Hill

**Affiliations:** 1 Leonard Davis School of Gerontology, University of Southern California, 3715 McClintock Ave, Los Angeles, CA 90089 USA; 2 Louisiana State University, Pennington Biomedical Research Center, Baton Rouge, 70808 LA USA

**Keywords:** Adipose Tissue, Senescence, FGF21, Lifespan, Metabolic health, Dietary restriction, Dietary protein, Amino acid

## Abstract

Cellular senescence and metabolic impairment occur during aging, with adipose tissue decline playing a key role in this process. Furthermore, the detriments of aging on adipose tissue function are further exacerbated by obesity. Dietary protein restriction (DPR), without reducing calorie intake, protects against age-related metabolic decline and extends lifespan through the metabolic hormone FGF21. Here, we demonstrate that protein restriction significantly decreases pro-oncogenic and senescence-related markers in adipose tissue, including SASP, *Cdkn1a Cdkn1a*, and SA-βgal staining. Additionally, mice fed a low-protein diet during diet-induced obesity demonstrated significant decreases in tumorigenic and cell cycle markers compared with mice fed a control protein and high-fat diet, suggesting that a low-protein diet decreases the burden of cellular senescence on adipose tissue in aged mice and aged obese mice. Conversely, mice lacking FGF21 failed to exhibit the benefits of protein restriction on markers of senescence in white and brown adipose tissue. These data demonstrate that protein restriction exerts distinct beneficial effects on white and brown adipose tissue remodeling on senescence and other markers associated with improvements in lifespan and particularly health span. Given the negative impact of cellular senescence on adipose tissue, protein restriction offers a potential dietary intervention to prevent the detriments of cellular senescence on adipose tissue function during obesity and aging.

**Supplementary Information:**

The online version contains supplementary material available at 10.1007/s11357-025-01853-w.

## Introduction

Aging contributes to the functional decline of adipose tissue, leading to poor health [1]. Thus, advancing in age and obesity induces an inflammatory environment within adipose tissue that is associated with an irreversible cell cycle arrest, also known as senescent cells (SnCs) [2]. Cellular senescence can be identified by (a) flattened morphology, (b) markers of cell cycle arrest, (c) a higher activity of β-galactosidase, and (d) production of senescent-associated secretory phenotypes (SASP) that include an array of cytokines, chemokines, matrix-remodeling proteases, and growth factors [3]. Together, SnCs and the associated inflammatory environment are detrimental to the generation of new adipocytes, damaging the physiological role of adipose tissue, thus impairing metabolic health and reducing lifespan [4].

Dietary interventions such as calorie restriction (CR), fasting mimicking diet (FMD), and dietary protein restriction (DPR) are confirmed to significantly improve health span and lifespan [5]. Furthermore, various gradient levels of CR in rodents have been shown to reduce the burden of SnCs in liver, adipose tissue, pancreas, and kidney [6]. Similar Outcomes in humans, such as in the CALERIE phase-II trial, observed that 14% CR for 2 years reduced SnCs and inflammasome effects in white adipose tissue [7]. DPR in the absence of energy restriction improves overall health and lifespan in non-human primates, rodents, fruit flies, and yeast by mechanisms that are independent of CR [8]. Also, human data suggest that lowering or altering the source of dietary protein content, as in the Okinawa diet, benefits health and lifespan [9], while high protein intake is associated with increases in all-cause mortality [10, 11]. Compared to CR, there is limited evidence on the SnCs and their related impact on metabolic health during DPR. A previous short-term feeding study in C57BL/6 male mice using varying dietary protein content showed that 3 months of feeding high-protein (30%) and combined high-protein (30%) and fat (60%) increased *p16* and *p21* mRNA levels in liver compared to a low-protein (10%) diet [12]. In this same study, mice fed a 10% low-protein diet compared to a 30% high-protein diet, despite high-fat or high-carbohydrate content in the diet, reduced hepatic *p53* expression [12]. Overall, these studies suggest DPR improves metabolic health and extends lifespan.

DPR triggers a series of adaptive behavioral and metabolic changes that are distinct from classic energy/calorie restriction paradigms. Many studies have emphasized the ability of protein or amino acid restriction to engage a host of cell-autonomous, intracellular nutrient-sensing pathways, including mechanistic target of rapamycin (mTOR), GCN2, AMPK, and autophagy [13]. Considering that the Liver is altered in response to nutrient cues, several years ago, it was discovered that the Liver-derived hormone fibroblast growth factor 21 (FGF21) serves as an effector signal of protein restriction, as such, the deletion of FGF21 blocks adaptive metabolic responses to PR in young mice [14]. Briefly, FGF21 is a 22 kDa endocrine hormone that is abundantly synthesized and secreted by the liver and signals for fibroblast growth factor receptors (FGFRs) and co-receptor beta-klotho (Klb) in downstream tissues [13]. FGF21 signaling alters adipose tissue and liver, improving energy expenditure, glucose metabolism, and upregulating the thermoregulatory marker UCP1 [15]. Our recent data indicate that FGF21/Klb signaling directly in the brain is essential for DPR to improve metabolic health and protect against diet-induced obesity in young mice [16]. The role and action of FGF21 also intersect with many pathways that regulate metabolism and aging. Mice that overexpress FGF21 have increased median survival by ~ 30% and 40% with a maximal Life extension in the 95th percentile, respectively, in males and females, compared to control littermates [17]. Within the notion of diet effects, aging, and hormonal regulation, additional studies from our lab demonstrated that FGF21 signaling during DPR is required to improve metabolic and kidney health, reduce frailty, preserve neuromuscular fitness, and extend lifespan in male mice [18]. Considering that aging and obesity are associated with profound changes in adipose tissue, and that adipose tissue contributes to energy expenditure and insulin sensitivity, we hypothesized that increases in FGF21 levels may mediate the beneficial effects of DPR on adipose tissue remodeling, reducing markers of senescence such as cyclin-dependent kinases (*Cdkn1a*, *Cdkn2a)*, senescence-associated beta-galactosidase (SA-βgal), and the various markers of the senescence-associated secretory phenotype (SASP).

Here, we leveraged adipose tissue (inguinal-WAT, epididymal-WAT, and interscapular brown adipose tissue-BAT) from a previous study in which C57BL/6 mice and *Fgf21* KO mice were fed a control or low-protein from 3–22 months of age [18], as well as two additional cohorts exposed to low-protein diet from 3–10 months of age or 16–22 months of age. We demonstrate that DPR reduces the burden of several senescence-related factors and genes that are likely to contribute to improvements in metabolic health during aging and obesity. We also show that LP-induced FGF21 action is critical to reduce systemic and local senescence-related inflammation in adipose tissue. Collectively, these data suggest that FGF21 plays a key role in the effects of protein restriction on adipose tissue remodeling and cell senescence-related effects. We propose that this adipose tissue remodeling is a key mediator of the improvements in metabolic health and lifespan.

## Methods

### Animals and diets

All animal procedures were approved by the USC Institutional Animal Care and Use Committee (IACUC) and the PBRC Institutional Animal Care and Use Committee (IACUC) and were performed following the guidelines and regulations of the NIH Office of Laboratory Animal Welfare. For studies at PBRC, male C57BL/6 mice (WT, Jackson Lab) were used in all studies. *Fgf21*-deficient mice on the C57BL/6 background (Fgf21-KO) were originally provided by Steven Kliewer (University of Texas Southwestern, Dallas, Texas, USA) and maintained as a colony at PBRC. Diets were formulated and produced by Research Diets as previously described [18] and were designed to be isocaloric by equally varying protein and carbohydrate while keeping fat constant. Normal-protein control diets (CON) contained 20% casein (by weight) as the protein source, while the low-protein diet (LP) contained 5% casein. High-fat diets (HF) also contained 20% casein (HFCON) and 5% casein (HFLP), respectively, but on a background of 60% fat. All diet compositions are provided in Supplemental Table 1. Mice that were clinically determined as being unable to thrive were removed from the study and euthanized. At the end of the study, mice were euthanized during the mid-light cycle in the fed state (unless otherwise noted) using acute exposure to CO2 followed by rapid decapitation. Mice were euthanized, and trunk blood was collected, centrifuged at 3000 × g, and serum collected. Tissues were collected and snap-frozen in liquid nitrogen for further analysis.

### Experimental design

#### Experiment 1: Effect of DPR on senescence-related factors in young to early-middle-aged mice with obesity

To investigate whether the early intervention of LP diet reduces the burden of senescence-related factors on metabolic health during aging and obesity, male C57BL/6 J mice were entered into the study at approximately 3 months of age and group-housed (4 per cage) at room temperature (23 °C). Mice were randomly assigned to one of 4 diets (10 mice/diet): normal-protein control (CON), LP, HFCON, or HFLP diet ad libitum (Supplemental Table 1). Bodyweight and food intake were recorded weekly throughout the experiment. Body composition was measured via TD-NMR (Bruker Minispec) at the start (3 months of age) and the end (10 months of age) of the study. Glucose homeostasis was evaluated by a glucose tolerance test at 9 months of age. After ~ 28 weeks of dietary manipulation, mice were euthanized at 10 months of age for blood and tissue collection for further processing and analysis. The experimental timeline (created by BioRender) is presented in Supplemental Fig. 1a.

#### Experiment 2: Effect of DPR on senescence-related factors during aging with obesity

**Fig. 1:**
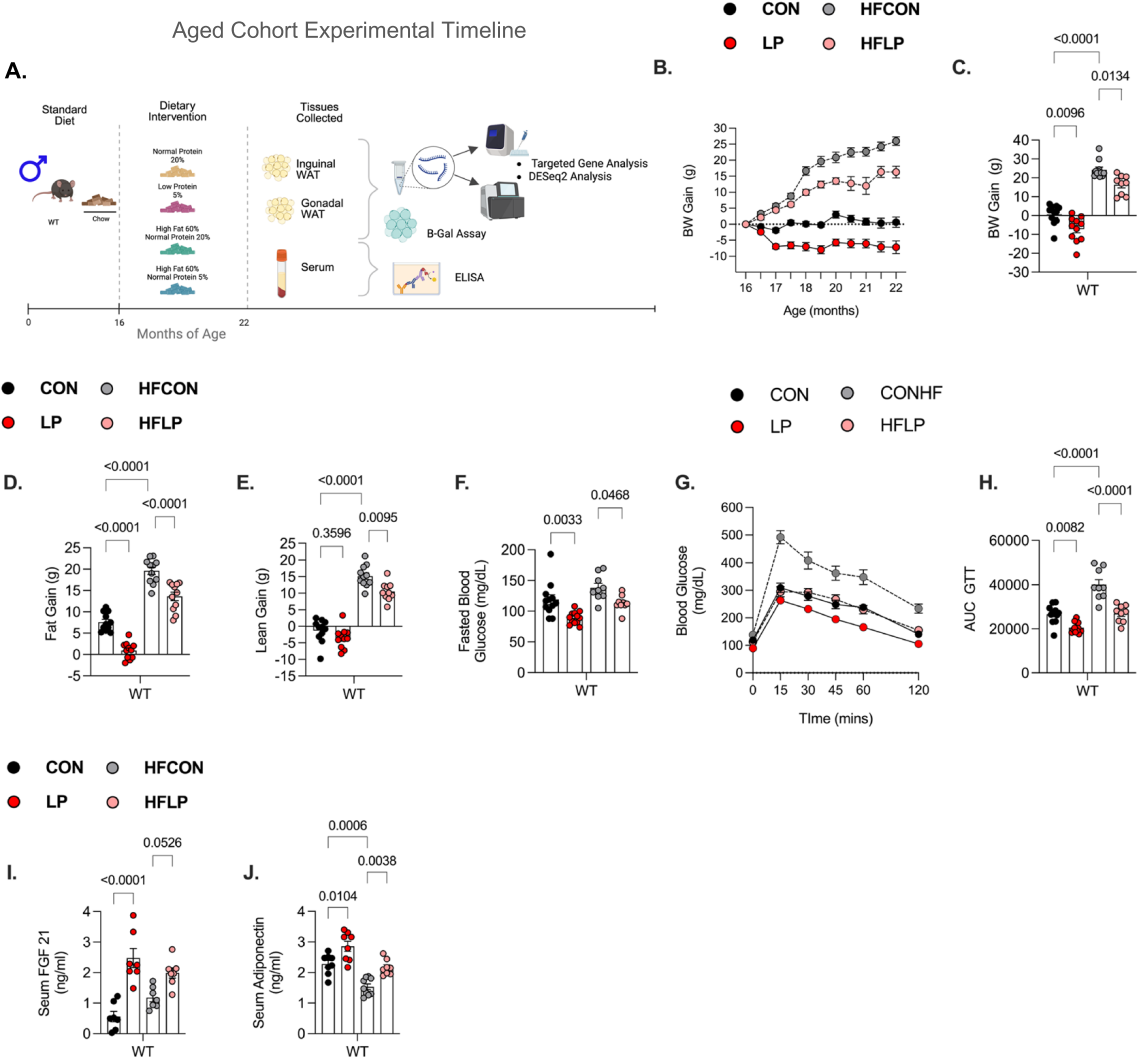
Low-protein diet late in life prevents age-related and obesity-related metabolic decline.** A** Graphical methodology: C57BL/6 (WT) male mice were placed on CON, LP, HFCON, and HFLP at 16 months of age (8–12 mice/group), various metabolic endpoints on BW and glucose homeostasis throughout the feeding phase of the study as indicated, and tissue collection at 22 m of age. **B** Body weight gain over time from initiation of diet. **C** Terminal body weight gain at 22 months of age. **D** Fat gain at 22 months of age. **E** Lean gain at 22 months of age. **F** Fasting blood glucose at 21 months of age. **G** Glucose tolerance test conducted at 21 months of age (*n* = 9 mice/diet). **H** Area under the curve glucose for the GTT. **I** Serum FGF21 levels at 22 months of age. **J** Serum adiponectin levels at 22 months of age. Statistical analyses were conducted using one-way ANOVA. All values are mean ± SEM, with significant main effects of protein or post hoc comparison within the fat*protein interaction

To examine the impact of age on dietary protein restriction-induced changes in senescence-related factors and metabolic Health, 16 months of age male C57BL/6 J mice were grouped-housed at room temperature (23 °C). Mice were assigned to either CON, LP, HFCON, or HFLP diets *ab libitum* for 26 weeks with body weight and food intake measured weekly. Body composition was measured via TD-NMR (Bruker Minispec) at the start (16 months of age) and the end (22 months of age) of the study. Glucose homeostasis was evaluated by a glucose tolerance test at 20 months of age. After ~ 24 weeks of dietary manipulation, mice were euthanized at 22 months of age for blood and tissue collection for further processing and analysis. The experimental timeline is presented in Fig. 1A.

#### Experiment 3: Role of fgf21 in mediated DPR-induced effects on senescence-related factors during aging

FGF21 is required to improve metabolic health and extend lifespan during prolong dietary protein restriction [18]. To expound on the role of FGF21 on senescence-associated phenotypes in the adipose, we leveraged stored inguinal white adipose tissue (iWAT), epididymal white adipose tissue (eWAT), brown adipose tissue (BAT), and liver from a previous study [18]. Briefly, male wildtype (C57BL/J6) and *Fgf21*-KO mice were entered into the study at approximately 3 months of age and group-housed (4 per cage) at room temperature (23 °C). Mice were placed on either CON or LP diet ad Libitum until 22 months of age (12 mice/diet/genotype). Mice were euthanized at 22 months of age, and adipose depots were used in this study to evaluate the role of FGF21 on markers and factors of senescence in aging mice. The experimental timeline is presented in Supplemental Fig. 4A.

### Glucose tolerance test (GTT)

Sixteen-hour-fasted mice underwent GTT by i.p. injection with 2 g glucose per kg of BW. Blood glucose levels were measured at 0, 15, 30, 45, 60, and 120 min via a handheld glucometer (Accu Check; Roche Diabetes Care, Inc. Indianapolis IN). The data for GTT are represented as mg/dL and as the area under the curve (AUC). Fasted glucose levels are reported at the time of GTT.

### Immunoassay determination of fgf21 and adiponectin

Serum concentrations of FGF21 (no. RD291108200R, Mouse and Rat FGF-21 ELISA, BioVendor) and adiponectin (#EZMADP-60 K, Mouse Adiponectin EMD Millipore Corporation) were determined via ELISA according to the manufacturer’s recommended protocol.

### Cellular senescence-associated beta-galactosidase (SA-b-gal) activity assay

Cellular SA-b-gal activity was adapted to inguinal White adipose tissue from 22 months of age C57BL/6 male mice and measured as previously described in whole adipose tissue explants [19]. Briefly, a small piece of iWAT fat was excised and fixed for 10 min 2% formaldehyde/0.2% glutaraldehyde (Sigma-Aldrich) dissolved in PBS at room temperature. Fat sections were then immersed in a rotated tubes full of freshly prepared SA-b-gal activity solution (1 mg/mL of X-gal, 40 mM citric acid/sodium phosphate at pH 6.0, 5 mM potassium ferrocyanide, 5 mM potassium ferricyanide,150 mM NaCl, and 2 mM MgCl2) at 37 °C in the dark. Photographs of iWAT samples among different groups were taken after 6 h of incubation and PBS rinse.

### Real-time PCR

RNA extraction and real-time PCR were conducted as previously described [18]. Adipose tissue depots (iWAT, eWAT, and BAT) of WT mice and *Fgf21* KO mice fed either normal protein (CON) or low protein (LP) = (*n* = 8–12 per group) were used for total RNA extraction using TRIzol reagent following the manufacturer’s protocol (Invitrogen), with the addition of a RNeasy Lipid Tissue Mini Kit (QIAGEN) for iWAT. RNA purity and quantity were determined by spectrophotometry using a Nanodrop One Spectrophotometer (Thermo Scientific™, Invitrogen). cDNA synthesis was performed with iScript (BioRad), and mRNA was quantified on the ABI 7900 platform using the ABI SYBR Green PCR Master Mix in optical 384-well plates (Applied Biosystems). Primer pairs were designed using the IDT RealTime qPCR Primer Design tool to span an intro-exon boundary (Supplemental Table 2). Target gene expression was normalized with cyclophilin as the endogenous control.

### Transcriptomic and bioinformatics analysis

Total RNA concentrations were diluted to 50 ng/ul. Total RNA-Seq libraries were constructed using Illumina’s Tru-Seq Stranded Total RNA Library Prep Kit with Ribo-Zero. RNA was sequenced on the Illumina NextSeq 500 using the High Output v2 Kit and paired-end sequencing forward and reverse reads (2 × 75 bp) with 75 million reads/sample. Bioinformatic analysis of RNA-seq data was conducted using the online platform using iDEP 2.01 [20, 21] which conducts differential expression and pathway analysis. In brief, raw read counts were imported into R version 4.2.1. for differential gene expression analysis using Bioconductor package DESeq2 version 1.46.0. to compare groups within the same adipose tissue type. DESeq2 genes with a false discovery rate (FDR) cutoff: *p*-value < 0.05 and log2 fold change > 1 were considered significant and differentially expressed. Functional analyses were performed with DEGs for both enrichment analyses and pathway analysis with PGSEA algorithm [22]; accordingly, differential results were used for PGSEA for Gene Ontology: Biological Process and KEGG (Kyoto Encyclopedia of Genes and Genomes—Database version release 104.0) to identify enriched pathways for diet and Gene interaction. A subset of differentially expressed Genes, top 30, were used for further visualization by heatmap plot.

### Quantification and statistical analysis

Data were analyzed using Prism by one-way, two-way, or repeated-measures ANOVA using the general linear model procedure. When experiment-wide tests were significant, post hoc comparisons were made using the LSMEANS statement with the PDIFF option and represent least significant differences tests for pre-planned comparisons. All data are expressed as mean ± SEM, with a probability value of 0.05 considered statistically significant.

## Results

### Low-protein diet prevents age-related and obesity-related metabolic decline

We recently reported that protein restriction induces metabolic benefits in middle-aged mice (diet initiated at 12 until 16 months of age) [18]. Here, we assess the impact of protein restriction during advanced aging to examine the impact of diet when most age-related detriments likely occur; thus, WT mice were fed either a CON (control-normal protein), LP (low-protein), HFCON (high-fat control), and HFLP (high fat–low protein) diet at 16 months until 22 months of age (*n* = 12 mice per diet) as outlined in Fig. 1A. Consistent with previous data middle-aged male mice, DPR at older age significantly reduced body weight gain in WT-LP mice compared to WT CON-fed mice (Fig. 1B and C). Initially, WT-HFLP-fed mice weight gain was parallel to WT-HFCON-fed mice, yet at 18 months of age, WT-HFLP-fed mice deviated from further increases in weight gain (Fig. 1B). Moreover, body weight gain in WT-CON and WT-HFLP mice was significantly reduced compared with WT-HFCON mice (Fig. 1 C). Body composition analysis shows that LP diet reduced both fat and lean gain in WT mice; however, the reduction in fat gain was greater in WT-HFLP mice compared with WT-HFCON mice (Fig. 1D). Our previous data show that the reduction in fat gain in middle-aged mice fed a high fat diet during DPR resembles similar gains in fat as aged counterparts fed a high fat diet [18]. However, in this cohort of aged mice, induction of DPR during obesity at older age significantly reduces body fat compared to high-fat feeding alone, as reflected in WT-HFLP mice compared to WT-HFCON mice (Fig. 1D). Interestingly, at older age, lean mass was not reduced in WT-LP mice when compared with their control WT-counterparts fed either CON. As expected, high-fat diet increased lean gain in both WT-HFCON and WT-HFLP-fed mice, although reductions in the lean gain of WT-HFLP mice were reduced compared with WT-HFCON mice (Fig. 1E).

In addition to the advanced aged cohort, we also investigated whether protein restriction reduces the burden of SnCs on metabolic health in early-middle age. Here, WT mice fed either CON, LP, HFCON, and HFLP diet from 3 to 10 months of age (*n* = 10 mice per diet). Metabolic endpoints were assessed throughout the feeding phase of the study as outlined in Supplemental Fig. 1a. At 10 months of age, BW gain was reduced by LP diet and the HFLP diet in WT mice compared with their control counterparts fed either CON diet or HFCON diet (Supplemental Fig. 1b and c). As such, BW gain in WT-HFLP mice resembled WT-CON mice, even though HFLP mice were consuming a 60% fat diet. As expected, HFCON diet increased BW gain compared to CON diet in WT mice (Supplemental Fig. 1c). Body composition analysis revealed that fat gain was reduced in WT-LP mice compared with WT-CON-fed mice, while HFCON diet increased fat gain compared to CON diet. Interestingly, fat gain in WT-HFLP mice was not different from WT-HFCON mice (Supplemental Fig. 1d). Lean gain was reduced in both WT-LP and WT-HFLP compared to their control diet counterparts; however, lean gain in WT-HFLP mice resembled lean gain of WT-CON mice (Supplemental Fig. 1e).

The metabolic function of adipose tissue declines during aging; furthermore, this decline is exacerbated by obesity [23]. Low protein and low amino acid fed mice have enhanced glucose clearance during a glucose tolerance test compared with their normal-protein fed counterparts; moreover, these improvements are correlated with increases in longevity-related circulating hormones [24]. To assess the impact of late in life DPR and DPR during diet induced obesity on glucose homeostasis, an intraperitoneal glucose tolerance test was conducted at 21 months of age. Glucose tolerance was significantly improved, as was fasted blood glucose, in WT-LP mice and WT-HFLP mice compared with WT-CON mice and WT-HFCON mice (Figs. 1 F and G). Shown by area under the curve, glucose clearance was similar in WT-HFLP mice and WT-CON mice; thus, HFLP diet normalized glucose homeostasis to CON diet effects, while HFCON diet worsened glucose clearance (Fig. 1H). This data indicates that DPR protects against glucose impairment during aging with obesity unlike the effects of high-fat diet as observed in WT-HFCON-fed mice. Similarly, fasting blood glucose and glucose tolerance were significantly improved in WT-LP mice and WT-HFLP mice as compared with WT-CON mice and WT-HFCON mice during middle age (Supplemental Fig. 1f-h).

To evaluate the effect of DPR on circulating hormones that are associtated to improved metabolic health and extended lifespan, mice were euthanized at 10 months and 22 months of age, and serum was used to determine FGF21 and adiponectin levels. Consistent with our and other reported data, DPR increases circulating FGF21 levels and adiponectin levels. A low-protein diet at early-middle and advanced age, even during obesity, increased FGF21 levels in both WT mice, compared to their control counterparts fed CON or HFCON (Fig. 1; Supplemental Fig. 1i). Adiponectin levels were also increased in both early-middle and aged LP-fed mice and HFLP-fed mice compared with their respective counterparts fed either CON or HFCON, while adiponectin levels were solely decreased by HFCON (Fig. 1 J; Supplemental Fig. 1j). These data suggest that older age and obesity during aging do not diminish the effects of a low-protein diet on increasing beneficial metabolic hormones FGF21 and adiponectin.

### Low-protein diet alters markers of senescence in adipose tissue

**Fig. 2:**
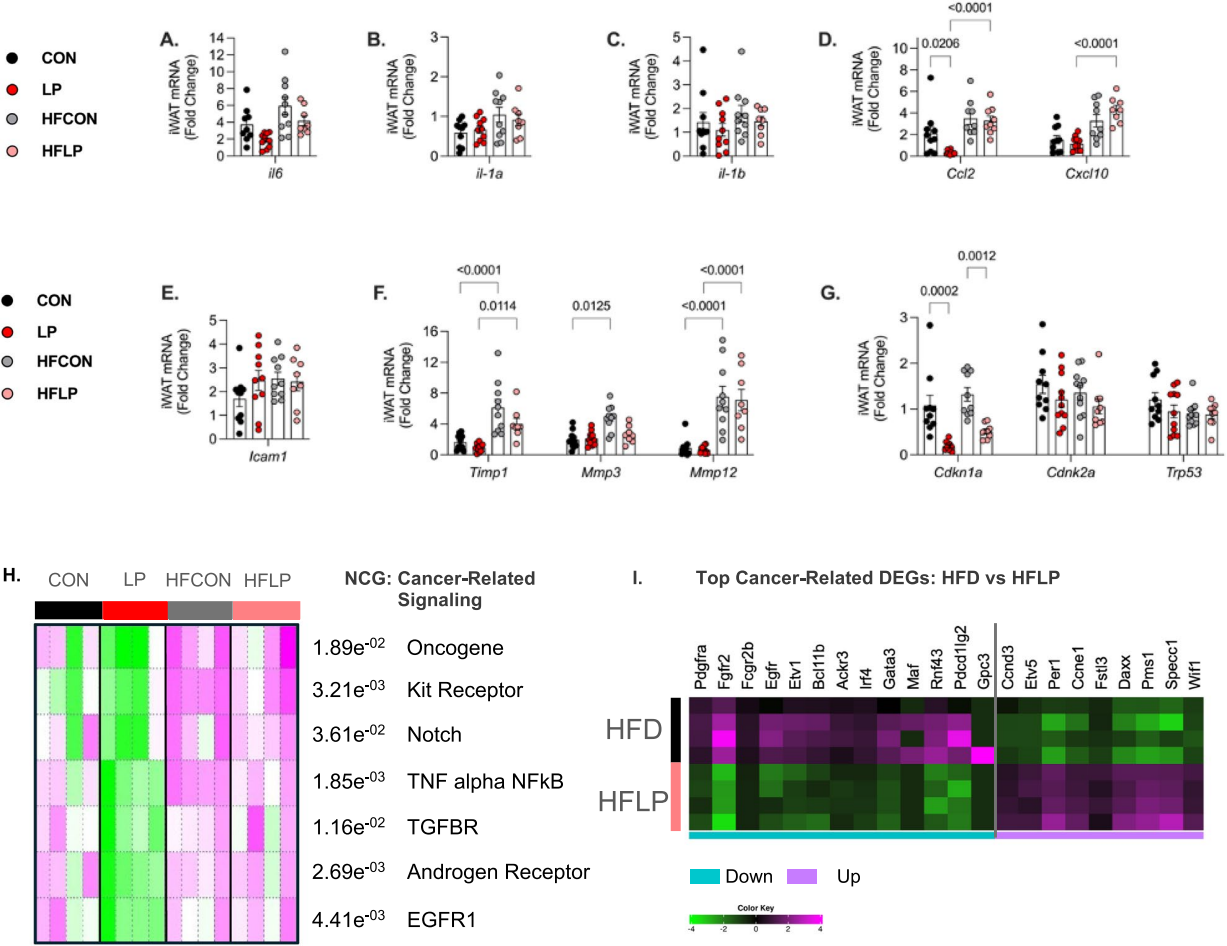
Low-protein diet reduces senescence-related markers in subcutaneous white adipose tissue (iWAT) during old age and aging with obesity. **A–H** Fold change of SASP-related markers in iWAT, interleukins (IL), monocyte marker, and chemoattract mRNA expression in iWAT at 22 months of age, *n* = 8 (CON), 10 (LP), 10 (HFCON), 10 (HFLP). **A** *Il6*. **B** *Il1a*. **C** *Il1b*. **D** *Ccl2* and *Cxcl10*. **E** Leukocyte adhesion molecule *Icam1*. **F** Matrix metalloproteinases *Timp1*, *Mmp3*, and *Mmp12*. **G** Cell cycle arrest *Cdkn1a* and *Cdkn2a* and tumor protein *Trp53*. **H** PGSEA: Parametric Gene Set Enrichment Analysis of cancer-related signaling from bulk-RNA seq. **I** Heatmap of top significant DEGs in HFD vs. HFLP fed WT mice. Statistical analyses were conducted using either a one-way or two-way ANOVA. All values are mean ± SEM, with significant main effects of protein or post hoc comparison within the fat*protein interaction

At the cellular level, white adipocytes are a large, single lipid droplet and play a major role to facilitate lipid storage and regulate energy and glucose metabolism and immune responses [25–30]. However, aging and obesity are associated with SnCs, inflammation, and local tissue remodeling that leads to metabolic impairment [31]. To extend on this concept, we investigated the impact of a dietary intervention of DPR on the burden of senescence-related markers in adipose tissue at older age. At 22 months of age, expression of markers of inflammation by host-defense interleukins *(Il6*, *Il1α*, and *Il1b*) or cell surface inflammatory markers (*Icam-1*) was not altered in the iWAT by either diet (Fig. 2 A, B, C, and E). However, LP diet altered the expression of markers of inflammation secreted by macrophages within adipose tissue; most notably, *Ccl2* was only reduced by LP diet; however, this HFLP diet increased *Ccl2* and *Cxcl10* (Fig. 2D). Likewise, markers of tissue remodeling by metallopeptidases including *mmp3*, *mmp12*, and *timp1* were only increased by high-fat diet, yet no impact of protein (Fig. 2 F). Moreover, cell cycle tumor suppressors *Cdkn2a* and *p53* expression were not altered; however, c*dkn1a* expression levels were significantly reduced by LP diet and HFLP diet in aged mice (Fig. 2G).

To capture the impact of diet with varying protein content on the transcriptional changes in adipose tissue during obesity and aging, we utilized an unbiased approach via bulk RNA-sequence analysis to expand on our targeted mRNA findings on other associated markers in iWAT. PGSEA heatmap clustering of all samples aligned to a curated collection of cancer genes, and associated drivers developed by the NCG:Network of Cancer Genes [32, 33] show that diets high in fat greatly increased in pathways associated with cancer-related signaling within our bulk-seq data set compared to LP diet (Fig. 2H). Interestingly, despite increased body weight gain and adiposity, HFLP-fed mice have similar measurements on improved glucose homeostasis via GTT compared with CON-fed mice. Therefore, we next explored the transcriptional changes in iWAT to expound on diet effects in aged WT mice fed either HFLP or HFD. DEseq2 analysis shows that iWAT from HFLP mice is enriched and downregulated in genes that are associated with the induction of pancreatic and bone cancers and lipomas (i.e., *Ackr3*, *Omd*, *and Rnf43*) and upregulated in genes that maintain proper regulation of the cell cycle and reduce the development of tumorigenesis through TGF-beta and WNT signaling (*Fstl3, Ccnd3, Per1, Daxx, Pms1 and Specc1 *) (Fig. 2I).

Studies show that starting CR at 12 months of age provides significant survival advantages in genetically heterogeneous mice [34–36]. And that CR promotes healthier biological phenotypes, specific reductions of markers of senescence in various mouse organs such as adipose tissue, intestine, and liver [37–39]. Therefore, we examined if DPR when initiated at 3 months of age until 10 months of age remodels adipose tissue to prevent age-related SnCs burden, hence 10 months of age represents a transitional period of when the onset of age-related illness typically occur. In the early-middle-aged cohort, at 10 of age months of age, expression of markers regarding inflammation by host-defense interleukins (*Il6*, *Il1a*, and *iI1b*) or immune cell-surface rolling adhesion marker *Icam-1* was not altered in iWAT by either diet (Supplemental Fig. 2a-d). However, LP diet reduced the gene expression of markers of inflammation secreted by macrophages within iWAT, most notably *Ccl2* and not *Cxcl10* (Supplemental Fig. 2e). Likewise, markers of repair of tissue damage and inflammation by metallopeptidases, notably *Mmp3*, were reduced by LP diet and HFLP diet, while *Mmp12* and *Timp1* trended lower expression but did not reach statistical significance (Supplemental Fig. 2f). Interestingly, markers of cell arrest and tumor suppressors C*dkn2a* and *Trp53* expression were not altered by protein or fat *x* protein interaction, yet C*dkn1a*, the gene that codes for *P21*, was significantly reduced by LP diet and HFLP diet (Supplemental Fig. 2g). Lastly, *Glb1*, *a* marker of increased lysosomal senescence, was reduced by LP diet and HFLP diet compared with respective control normal-protein and high fat normal-protein diet (Supplemental Fig. 2h).

In epididymal adipose tissue (eWAT), diets high in fat content altered markers of chemotaxis and tissue remodeling in both early-middle age and aged WT mice. Here, *Ccl2* and *Timp1* expression levels were significantly increased during diet-induced obesity, whereas LP diet significantly reduced *Timp1* levels relative to CON diet and diets high in fat (Supplemental Fig. 2i and 2j). Also, LP diet and HFLP reduced the expression of multiple cell-cycle related senescence markers, most notably *Cdkn1a*, while only HFCON increased *Cdkn2a* expression compared to either CON, LP, or HFLP diet in WT mice (Supplemental Fig. 2k and l). Lastly, Glb*1* gene expression levels were not altered by varying dietary protein content, although higher dietary fat content significantly increased levels in early-middle-aged WT mice (Supplemental Fig. 2m).

**Fig. 3:**
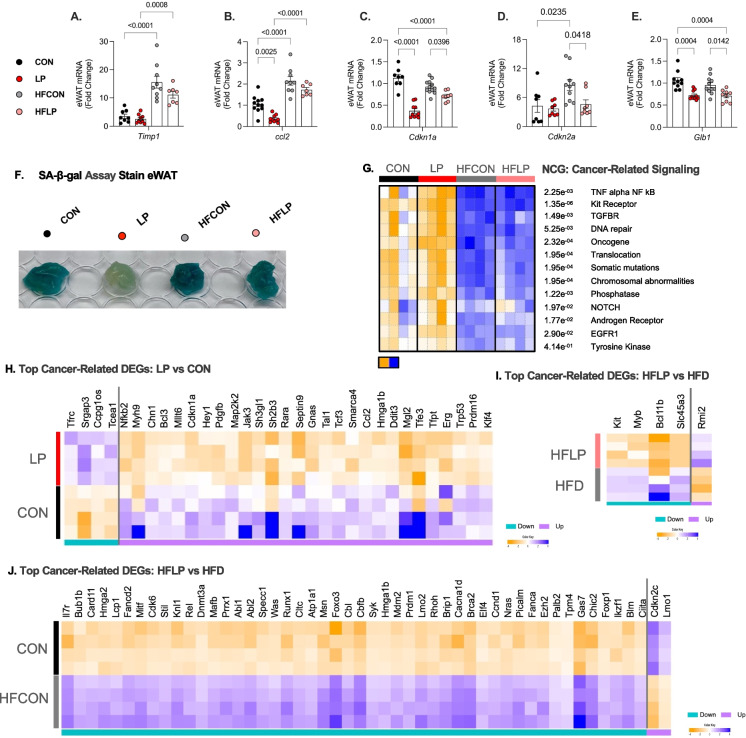
Low-protein diet reduces senescence-related markers in epidydimal adipose tissue during old age and aging with obesity.** A–E** Fold change of markers of SASP-related, cellular arrest, cell proliferation, and monocyte chemoattract mRNA expression in visceral (eWAT) adipose tissue of WT mice at 22 months of age, *n* = 8 (NP), 10 (LP), 10 (HFCON), and 10 (HFLP). **A** *Timp1*. **B** *Ccl2*. **C** *Cdkn1a*. **D** *Cdkn2a*. **E** Lysosomal beta-D-galactosidase *Glb1*. **F** Senescence-associated β-galactosidase staining in explant of eWAT per diet. **G** PGSEA: Parametric Gene Set Enrichment Analysis of cancer-related signaling from bulk-RNA seq. **G** Heatmap of top 30 DEGs in CON and LP fed WT mice.** H** Heatmap of top 5 DEGs in CON and HFF fed WT mice.** J** Heatmap of top 50 DEGs in CON and HFLP-fed WT mice. Heatmaps are listed by FDR < 0.05, color key shows logFC_2_ heatmaps. Teal blue represents downregulated, and lavender represents upregulated enriched genes within the comparison groups. Statistical analyses were conducted using two-way ANOVA. All values are mean ± SEM, with significant main effects of protein or post hoc comparison within the fat*protein interaction

Similarly, as observed in 10-month-old mice, in 22 months of age, *Timp1* and *Ccl2* expression levels were increased most notably by high-fat diet (Fig. 3 A and Fig. 3B). Yet, in aged mice, LP diet reduced chemotactic factor *Ccl2* expression when compared to CON, HFD, and HFLP-fed mice (Fig. 3B). As observed in the early-middle-aged cohort, LP and HFLP diet reduced the expression of multiple senescence markers in eWAT in 22-month-old mice, particularly C*dkn1a*, *Cdkn2a*, and *Glb1* (Fig. 3C–E). Additional identification on whole adipose tissue using a functional enzymatic assay to detect β-galactosidase activity shows that LP diet prevented the occurrence of lysosomal hydrolase, thus the cleavage of the terminal β-d-galactose residues in iWAT (Fig. 3 F).

We also conducted transcriptomic analysis of eWAT from this same cohort of aged mice fed either NP, LP, HF, or HFLP to identify the molecular pathways through which DPR reduces markers of senescence and related-SASP during aging and obesity. Correspondingly, as in iWAT, diets high in fat increased NCG:Cancer related pathways in aged mice, though with lesser extent in CON-fed mice in eWAT compared to LP-fed mice (Fig. 3G). DESeq2 analysis shows that CON diet increased gene expression of many other senescence-related and cancer-related markers (i.e., *Nkkb2*, *Myh9*, *Jak3*, and *Tfe3*) and reduced in genes associated with DNA stability and cell cycle (*Srgap3*, *Ccpg1os*, *Tecea*) compared to LP diet (Fig. 3H). Interestingly, not many cancer-related genes were significantly different in HFLP-fed mice compared to HFD mice; thus, only 5 genes (4 downregulated and 1 upregulated) were espressed. Here, in 22 months of age, HFLP mice had lower expression of pro-oncogenic and nutrient transporter signatures (*Myb*, *Bcl11b*, *Kit*, and *Slc45a3*) and increased expression of *Rmi2* (-recQ-mediated Genome instability 2), a gene identified in pathways associated with maintaining genome stability (Fig. 3I). And lastly, HFD was associated with rampant activation of immunosenescent- and cancer-related genes (*Hmga1b (2a)*, *Gas7*, *Foxo3*, *Braca2*, and *Lmo2)* (Fig. 3 J). Together, these data in iWAT and eWAT provide evidence that DPR during middle and advanced age reduces multiple markers of cell cycle arrest and even more reduces pro-oncogenic markers. Furthermore, the increase of senescence-related markers is dependent on adipose tissue location and age in conditions of obesity.

### Fgf21 is required to remodel distant signatures in adipose tissue on thermogenesis, senescence, and peripheral vascular signaling in aged mice during DPR

**Fig. 4:**
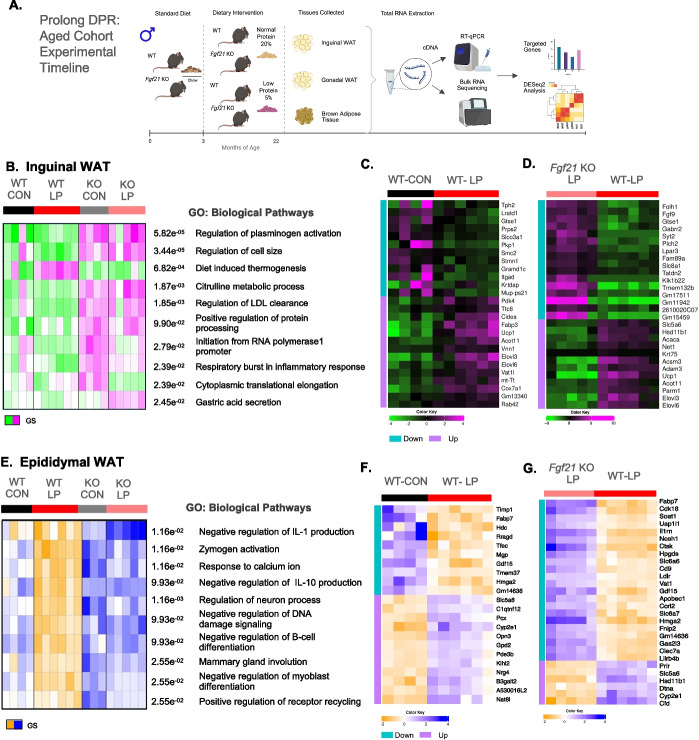
Low-protein diet induces FGF21-dependent transcriptional signatures on improved thermogenesis and reduced markers on cell senescence in white adipose tissue. Inguinal WAT and epididymal WAT, from a previous study, were used to perform bulk RNA-seq from C57BL/6 (WT) and *Fgf21* KO mice fed either normal-protein control (CON) and low-protein (LP) diet at 3 months of age until 22 months of age. **A** Graphical methodology: subcutaneous (iWAT), visceral (gWAT), and brown (BAT) adipose tissue from a previous study of C57BL/6 (WT) and *Fgf*21 KO mice fed either normal-protein (CON) or low-protein (LP) at 3 months of age until 22 months of age. **B–D** Inguinal WAT. **B** PGSEA: Parametric Gene Set Enrichment Analysis for GO: Biological Processes. **C** Heatmap of top DEGs in CON and LP fed WT mice. **D** Heatmap of top DEGs in LP-fed WT mice and *Fgf21* KO mice.** E–G** Epididymal WAT.** E** PGSEA: Parametric Gene Set Enrichment Analysis for GO: Biological Processes. **F** Heatmap of top DEGs in CON and LP fed WT mice. **G** Heatmap of top DEGs in LP-fed WT mice and *Fgf21* KO mice. Heatmaps are listed by FDR < 0.05, and color key shows logFC_2_ heatmaps. Teal blue represents downregulated, and lavender represents upregulated enriched genes within the comparison groups

The data above demonstrate that diets low in protein reduce some markers of senescence in adipose tissue during aging and obesity. Moreover, these data are associated with increases in FGF21. We recently reported that FGF21 is required to improve metabolic health, preserve physical fitness, reduce frailty, and extend lifespan during lifelong dietary protein restriction in male mice [18]. Our recent data confirmed that LP-fed WT mice live longer than CON-fed WT mice and FGF21 KO mice (whole-body *Fgf21* deficient) fed either CON or LP diet. Also in this same study, FGF21 KO LP-fed mice have reduced lifespan compared to their CON-fed littermates. To further investigate this conundrum on reduced lifespan and the related declines in energy and glucose homeostasis the setting of FGF21-signaling deficiency, we assessed the effect of protein restriction on transcriptome remodeling in adipose tissue. Here, we leverage liver, subcutaneous, visceral, and brown adipose tissue from Our previous aging and Lifespan study, a cohort of 22 months of aged WT and FGF21 KO mice fed either CON or LP diet (Fig. 4 A) [18], to perform targeted gene analysis and an unbiased approach using bulk-RNA-seq (Supplemental Fig. 3) to provide insight regarding genotype and diet interaction on markers of metabolism, immune function, and senescence.

To identify the necessity of FGF21 signaling on reducing senescence, we first targeted for general markers in White adipose tissue depots. In iWAT of 22-month-old mice, LP diet trended to reduce macrophage marker *Cd68* (cluster of differentiation 68) and *Ccl2* (also known as monocyte chemoattractant protein-1) compared with CON diet in WT mice and FGF21 KO mice fed either diet, yet these trends did not reach statistical significance (Supplemental Fig. 4.1a). Also, LP diet reduced *Cdkn1a* compared to CON diet in WT mice and *Fgf21* KO mice fed either diet (Supplemental Fig. 4.1b). Furthermore, tumor suppressor genes *Cdkn2a* and *Trp53* seemed to have higher expression during LP diet in both WT and *Fgf21* KO mice; however, these levels of expression did not reach significance (Supplemental Fig. 4.1c and d). Similar as in iWAT, in eWAT, *Ccl2* was reduced by diet effects protein and dependent on FGF21 (Supplemental Fig. 4.1d). Interestingly, anti-inflammatory response markers associated to improved insulin sensitivity and macrophage-2 phenotypes are enhanced by LP diet and dependent on FGF21. Thus, LP diet in WT mice increased *Cd206* and *Pnpla2* (also known as ATGL) gene expression compared to WT-CON and FGF21 KO mice fed either diet (Supplemental Fig. 4.1f and g). These data suggest that LP-induced FGF-21 signaling is required to reduce macrophage recruitment and the occurrence of cell-cycle arrest, in turn, to preserve adipose tissue function during aging.

To capture the transcriptional changes in adipose tissue on senescence and metabolic-related factors, we utilized an unbiased approach via bulk RNA-sequence analysis to expand on our targeted mRNA findings on *Cdkn1a* and related markers of senescence shown in Supplemental Fig. 4.1. In addition, to gain functional insight on DEGs, parametric analysis of gene set enrichment (PGSEA) method with all groups was performed. Briefly, this method leverages individual sample data of expressed genes that are then aligned to annotation sets for Gene Ontology (GO: Biological Process) and Kyoto Encyclopedia of Genes and Genomes (KEGG).

Here, in iWAT, PGSEA Heatmap clustering of all samples shows that only diet-induced thermogenesis was notably increased among the top 10 pathways and exclusive to WT-fed LP mice (Fig. 4B). The most relevant biological processes are associated with lipid regulation including blood clot degradation, low-density lipoprotein clearance, and inflammatory responses. Interestingly, these pathways were mostly increased in *Fgf21* KO mice with some effects by diet (Fig. 4B). To connect the observed improvements in metabolic health during DPR, we next sought out to identify a more comprehensive remodeling of in iWAT by diet effects and *genotype x diet* interaction effects. DESeq2 Heatmap clustering revealed that the top 30 genes expressed are enriched and upregulated in thermogenesis, TCA cycle, and fatty acid metabolic processes (i.e., *Acot11*, *Ucp1*, *Cox7a1*, *Fabp3*, *Elvol3*, and *Elvol6*) and downregulated in genes associated with positive regulation of receptor binding in tumor growh and immune cell signaling (i.e., *Gtse1*, *Lratd1*, *Itagd*, *Folh1*, *Fgf9*, *Tmem132b*) in WT LP-fed mice relative to WT CON-fed mice and *Fgf*21KO LP-fed mice, respectively (Fig. 4C and D; supplemental data file).

The location of visceral white adipose tissue (vWAT) and metabolic activity, like this, the adipose tissue in the has a higher proportion of resident macrophages, particularly those with a pro-inflammatory M1 phenotype, breaks down and mobilizes lipids leading to increased accumulation into vital organs, promoting inflammation and metabolic disease [40, 41]. For eWAT, a total of 956 genes were downregulated and 718 genes were upregulated in response to varying dietary protein content feeding in WT and *Fgf21* KO mice at 22 months of age as shown by the Venn Diagram (Supplemental Fig. 4.2b). PGSEA heatmap clustering of all samples shows that only LP-fed WT mice had reduced gene expression in biological processes associated with immune regulatory responses such as IL-1 production, negative regulation of DNA damage signaling, and B-cell differentiation (Fig. 4E). We next compared *diet x genotype* in eWAT to gain insight on the complete remodeling effect of dietary protein on adipose tissue to connect our *Cdkn1a* qPCR data and related markers of immuno-senescence with Our bulk-RNA seq data. Heatmap clustering shows the top 30 genes expressed are enriched and mostly downregulated in immune activation processes such as cytokine and phagosome complement cascades (i.e., *Timp1*, *Fabp7*, *Hdc*, *Mgp*, *Hmga2*, *Gdf15*, *Fnip2*, *Vat1*, and *Lilrb4b*) and upregulated in insulin signaling, lipid metabolism, and cytochrome-mediated processes (i.e., *Nrg4*, *C1qtnf12*, *Opn3*, *Cyp2e1*, *Hsd11b1*, and *Cdf*) in WTLP-fed mice relative to WTCON-fed mice and *Fgf21*KOLP-fed mice, respectively (Fig. 4F and G: supplemental data file).

**Fig. 5:**
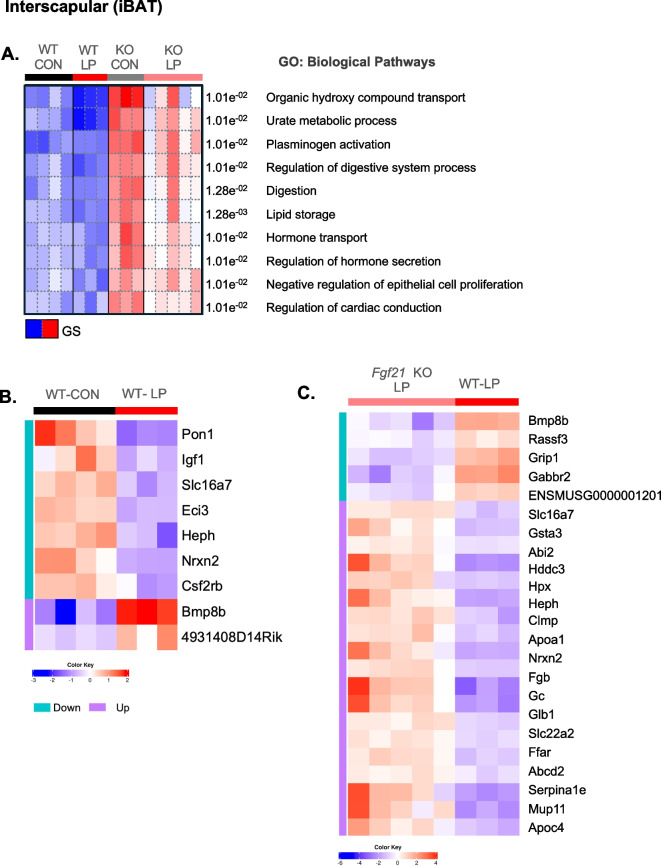
Low-protein diet induces fgf21-dependent transcriptional signatures on improved vascular-related remodeling in brown adipose tissue. BAT from a previous study was used to perform bulk RNA-seq from C57BL/6 (WT) and *Fgf21* KO mice fed either normal-protein control (CON) and low-protein (LP) diet at 3 months of age until 22 months of age. **A** PGSEA: Parametric Gene Set Enrichment Analysis for GO: Biological Processes.** B** Heatmap of top DEGs in CON and LP fed WT mice. **C** Heatmap of top DEGs in LP fed WT mice and *Fgf21* KO mice. Heatmaps are listed by FDR < 0.05, and color key shows logFC_2_ heatmaps. Teal blue represents downregulated, and lavender represents upregulated enriched genes within the comparison groups

Early work of Robert Emrie Smith in 1962 demonstrated that brown adipose tissue generates heat to maintain body temperature through non-shivering mechanism. This pioneering work led to new discoveries that identify the role of BAT in regulating energy and metabolic balance through sympathetic and sensory processes [42]. In the context of dietary protein, other studies show that reduced protein intake activates AgRP (agouti-related peptide) and NPY (neuropeptide Y) neurons on energy balance including thermogenesis in the hypothalamus [43]. To expand on this concept, we recently published that FGF21-signaling in the brain is required to alter feeding behavior and enhance thermogenesis health in young mice [16]. Therefore, we next investigated the role of BAT on transcriptional signatures that Likely contribute to improved Health during DPR. Here, in BAT, a total of 352 genes were down- and 59 genes were upregulated in WT and *Fgf21* KO mice fed either CON or LP diet at 22 months of age as shown by the Venn Diagram (Supplemental Fig. 5a). PGSEA clustering visualized that most GO: biological processes are primarily influenced by genotype. Likewise, WT compared to *Fgf21* KO mice, on changes in regulating of lipid storage, cardiac conduction, hormonal secretion, and transport compared to varied protein intake (Fig. 5 A). Interestingly in BAT, LP diet increased gene expression of many peripheral vascular biological processes associated with energy regulatory proteins that synchronize cell to cell communication between neuronal junctions (synapses) and nerve cells (i.e.*, Grip1*, *Bmp8b*, *Gabbr2*) and reduced gene expression of oxidative stress and lipotoxicity (i.e.*Pon1, Eci3, Hddc3, Sperina1e, and Apoc4*) compared to CON-fed WT (Fig. 5B; supplemental data file) and *Fgf21*KO (Fig. 5C; supplemental data file) mice fed either diet at 22 months of age.

In general, our targeted gene expression and bulk-RNA Seq data in WAT depots and BAT indicate that adipose tissue adapts to a low-protein induced FGF-21 signaling to remodel the transcriptome to reduce the burden of senescence and related markers. In iWAT, the most relevant KEGG pathways are associated with cancer, infection, and stress such as basal carcinoma, salmonella infection, and Cushing syndrome. And also, these pathways are more abundant in *Fgf21* KO mice fed either diet (Supplemental Fig. 4.2c). Secondly, eWAT was associated with tumor suppressor pathways and ion regulation such as glioma, p53 signaling, and aldosterone regulation of sodium. Interestingly, these pathways were significantly reduced only in WT-LP-fed mice when compared to WT-CON-fed mice and *Fgf21* KO mice fed either CON or LP diet (Supplemental Fig. 4.2d). And lastly in BAT, at 22 months of age, *Fgf21* KO mice fed CON diet were mostly enriched in KEGG pathways of amino acid metabolism, chemical carcinogenesis, and metabolism in response to prostaglandins relative to *Fgf21* KO LP-fed mice and WT mice fed either diet (Supplemental Fig 5b). Together, these data indicate that the beneficial role of LP-induced FGF21-signaling on metabolic health relies on synergistic inputs that are facilitated by thermogenic and inflammatory profiles in the subcutaneous and visceral adipose tissue.

## Discussion

Dietary strategies that reduce caloric intake, restrict the time of food intake, or adjust macronutrient composition have been shown to modulate molecular and cellular pathways that protect against age-related diseases [5]. Among the various tissues affected by dietary restriction, adipose tissue has earned significant attention for its central role in energy and glucose homeostasis, inflammation, and metabolic health [44–47]. Consequently, the biological detriments of aging and obesity share overlapping molecular signatures in adipose tissue, most notably a chronic pro-inflammatory state. This inflammation impairs preadipocyte proliferation and differentiation, thereby promoting cellular senescence in mature adipocytes, leading to altered hormone secretion and actions, such as insulin, adiponectin, and leptin, further inducing SnCs in adipose tissue [2, 3, 23, 48].

Studies in both young and aged mice have shown that the endocrine hormone fibroblast growth factor 21 (FGF-21) is required for sensing and adapting to low-protein diets to improve metabolic flexibility, enhance neuromuscular function, reduce frailty, and extend lifespan [18, 49]. Here, we show that low protein-induced FGF21 expression at both early-middle and advanced age protects against the burden of senescence-related remodeling in adipose tissue and preserves metabolic health during aging and diet-induced obesity.

### Low-protein diet at older age and during obesity benefits metabolic heath

Previous studies have shown that dietary protein restriction improves cardiovascular and metabolic health in humans and non-human primates and enhances health-span and lifespan in mice [8, 49]. Likewise, these data reflect the impact of protein restriction and individual amino restriction on metabolic health. Our new findings confirm that LP diets reduce body weight and fat gain, improve glucose clearance, and lower fasting glucose levels when interventions are initiated in both early-middle-aged and older-aged mice. These effects are also maintained under conditions of high-fat feeding; thus, WT mice fed the high-fat, low-protein (HFLP) diet showed reduced body weight, fat accumulation, and improved glucose tolerance compared to high-fat control (HFCON) counterparts.

Preserving lean mass during aging is critical for reducing sarcopenia risk [51]. While CR and DPR can lead to lean mass loss, the extent depends on macronutrient composition [46]. In our study, LP-fed WT mice maintained lean mass despite reductions in fat mass when dietary intervention began later in life. At 22 months of age, HFLP diet reduced total lean mass; however, this difference was only comparable to the increased lean mass in HFCON mice. We suggest that this finding is attributed to the general reduced growth of HFLP mice and not of malnourishment due to improved metabolic profiles compared to HFCON-fed mice.

### Low-protein diet reduces senescence burden and extracellular matrix remodeling in white adipose tissue during aging

Aging is associated with the accumulation of senescent cells and increased risk for cancer and metabolic dysfunction. In this study, we show that dietary protein restriction (DPR) modulates key molecular pathways involved in cellular senescence, inflammation, and extracellular matrix (ECM) remodeling within white adipose tissue (WAT) during aging and diet-induced obesity. Specifically, we observed that low-protein diets reduced the expression of canonical senescence markers, including Cdkn1a (p21) and Cdkn2a (p16), in both inguinal (iWAT) and epididymal (eWAT) fat depots. While cellular senescence serves as a tumor-suppressive mechanism, its chronic activation promotes the senescence-associated secretory phenotype (SASP), driving inflammation, tissue dysfunction, and age-related disease [50–53]. Thus, mitigating senescence via dietary intervention may help maintain adipose tissue homeostasis across the lifespan.

Although the metabolic impact of DPR on metabolic health has been well studied [10, 54], data linking tissue-specific response to low-protein diets on reduced senescence remain limited. Our findings align with a recent study showing that short-term DPR decreased hepatic gene expression of key SASP markers—IL-6, IL-1α, IL-1β, TNF-α, CXCL1, CXCL10, PAI-1, and CCL2—regardless of dietary fat content [12]. In our study, we found that Ccl2, a chemokine responsible for macrophage recruitment, was significantly reduced in iWAT of both middle-aged and aged mice fed a low-protein diet. In contrast, pro-inflammatory interleukins (IL-6, IL-1α, IL-1β) and ICAM1 were not significantly altered by dietary fat or protein content, suggesting that diets low in protein may selectively influence chemotactic signaling more than interlukin expression in adipose tissue.

Inflammatory signaling in adipose tissue regulates the turnover of the extracellular matrix through tissue inhibitors of metalloproteinases (TIMPs) and matrix metalloproteinases (MMPs). TIMPs inhibit MMP activity and contribute to homeostatic tissue remodeling under physiological conditions, whereas dysregulation of metalloproteinases are observed in obesity, fibrosis, and cancer [55–58]. Studies have shown that *Timp1* functions as a precursor to protect local tissue from the development and progression of inflammation and tumorigenesis during diet-induced obesity in mice [55, 59]. Other studies have demonstrated that the expression of MMPs (MMP-2, MMP-3, MMP-12, MMP-19, MMP-14) and TIMP-1 is upregulated in adipose tissue during high-fat diet and that deficiency of *Timp1* exacerbates the effects of MMPs during diet-induced obesity [57]. We observed that diets high in fat increased the expression of Timp1, Mmp3, and Mmp12 in both subcutaneous and visceral adipose tissue. Notably, the effects of low-protein diets were more prominent in visceral adipose tissue, where they suppressed these markers of matrix remodeling. Our findings are consistent with studies demonstrating that TIMP-1 expression increases in WAT during high-fat feeding and that TIMP1 deficiency exacerbates MMP-mediated tissue degradation during obesity [55, 58, 60]. Additionally, our data suggest that low-protein induced-FGF-21-dependent thermogenic pathways, may play a fundamental role in reducing ECM remodeling activity in aged adipose tissue.

In addition to suppressing inflammation and matrix remodeling, low-protein diets reduced genes linked to regulation of the cell division. Like this, *Glb1*, a gene encoding senescence-associated β-galactosidase, was significantly reduced in LP- and HFLP-fed mice compared to controls. Moreover, iWAT from HFLP mice exhibited increased expression of gene profiles that are suggestive of reduced lipoma formation and decreased cellular proliferation. Although lipomas are benign, they are often associated with stem cell dysfunction and chronic low-grade inflammation [61]. Notably, expression of *Rnf34* and *Fgfr2* was reduced in iWAT from HFLP mice. RNF34 is an E3 ubiquitin ligase that targets apoptotic proteins (e.g., caspases) and has been implicated in lipid metabolism and tumor progression [62, 63]. Reduced RNF34 expression may reflect a downshift in pro-oncogenic signaling and represent a potential protective mechanism by which DPR limits abnormal cell growth and inflammation in adipose tissue during aging.

Interestingly, in eWAT, we observed an increased expression of *Rmi2* (RecQ-mediated Genome instability protein 2) in response to dietary protein restriction. RMI2 is a critical component of the BLM–Topoisomerase IIIα–RMI1/2 (BTR) complex, which safeguards genome integrity by promoting homologous recombination and suppressing illegitimate DNA exchanges [64]. The function of this complex becomes increasingly important with age, as bloom expression levels decline, leading to increased DNA damage, mitochondrial dysfunction, and senescence [64–68]. Thus, increased *Rmi2* expression in aged protein-restricted mice may represent a compensatory mechanism to maintain genomic surveillance and support long-term tissue resilience.

Taken together, these findings suggest that low-protein diets reduce the burden of senescence and limit ECM remodeling in both subcutaneous and visceral adipose tissue during aging. Importantly, the beneficial effects of DPR were evident even when initiated at older ages, supporting the potential for late-life dietary interventions to improve adipose tissue function and systemic metabolic health.

### Role of fgf21 on markers of senescence in aging adipose tissue

There is substantial evidence that subcutaneous inguinal (iWAT) and visceral (vWAT) adipose depots play critical roles in metabolic regulation and immune responses; however, their molecular functions are distinct [41, 69]. Aging and obesity contribute to the functional decline of these tissues and are associated with increased immune activation, leading to the senescence-associated secretory phenotype (SASP) and cell cycle arrest in adipose tissue [2, 19, 70]. For example, various immune cells within adipose tissue, including resident macrophages (adipose tissue macrophages, ATMs), regulate lipid uptake and storage to maintain metabolic homeostasis and insulin sensitivity [71]. However, chronic activation of ATMs promotes SASP, leading to DNA damage, extracellular matrix degradation, and impaired adipogenesis. In this part of the study, we sought to further explore transcriptomic remodeling of iWAT, epididymal WAT (eWAT), and brown adipose tissue (BAT) in response to dietary protein restriction. We also aimed to define the role of FGF21 signaling in regulating senescence-related gene expression by comparing wild-type and FGF21 knockout (KO) male mice at 22 months of age.

Our initial approach using targeted quantitative-PCR identified makers of SASP and cell arrest in iWAT and eWAT. In iWAT, LP diet reduced gene expression levels of *Cdkn1a* compared to CON-diet. We also observed that markers of functional health in visceral adipose tissue. Triglycerides must be broken down, mobilized, and used for cellular energy and are in part regulated by Lipolytic enzymes such as patatin-like phospholipase domain-containing protein 2—*Pnpla2* (also known as adipose triglyceride lipase). Furthermore, lack of *Pnpla2* in rodent models has been shown to produce obesity, adipose inflammation, and impaired insulin signaling [69], while overexpression of *Pnpla2* protects against the metabolic insults of diet-induced obesity [32]. In eWAT, LP-induced FGF21-signaling was more associated with genes that induced anti-inflammatory markers *Cd206* and *Pnpla2*, and this finding was correlated with reduced *Ccl2* expression.

We continued to investigate the role of FGF21 using an unbiased approach of bulk RNA sequencing in iWAT, eWAT, and BAT to determine if FGF21 deficiency modulates cellular senescence during prolonged protein restriction in WT and *Fgf21* KO mice. In comparison, DEGs in subcutaneous iWAT were mostly enriched in upregulated pathways driving lipid metabolism, while DEGs in visceral eWAT were mostly enriched in upregulated pathways related to immune and viral processes. Consistent with our previous data and others in iWAT, thermogenic and metabolic genes *(Ucp1*, *Cidea*, *Elvol6*, *Elvol3*, *Acsm3*) are increased and among the top 30 expressed genes in LP-fed WT mice compared to CON-fed WT and *Fgf2*1 KO mice fed either diet. And although we have not in the past fully identified the transcriptomic remodeling of protein restriction in eWAT, we here show that SASP-related genes (*Gdf15*, *Ccl2*, *Cdk18*, *Lilrb4b*, *Hmga2*) are reduced by LP-diet in WT compared to CON-diet in WT mice and that this reduction in SASP-related genes is dependent on low-protein induced-FGF21-signaling. Furthermore, at 22 months of age, LP diet reduced proinflammatory tissue remodeling genes such as *Timp1* in eWAT compared to CON diet in WT mice, and these findings were FGF21 dependent. Interestingly, in LP-fed *Fgf21* KO, the top 30 genes in eWAT showed reduced expression of genes that facilitate heart and skeletal muscle development function (i.e., *Dtna*) and *Cyp2e1* which encodes the enzyme cytochrome P450 that metabolizes drugs and other systemic toxins compared to WT-LP-fed mice. We suggest that this finding in eWAT is likely associated with the declines in neuromuscular function, such as decreases in grip strength and rotarod latency, in *Fgf21* KO mice that occur during protein restriction [18].

Brown adipose tissue is unique to WAT regarding total volume in humans; thus, in adults, it is estimated to be 0.05–0.1% of total body mass [70]. BAT phenotypically has smaller lipid droplets compared to WAT but shares similar roles to mobilize energy and can be activated by cold induction. Our earlier data show that low protein-induced FGF21 drives thermoregulation and energy expenditure independent of LP-induced metabolic measures such as body weight [71, 72]. Hence, deletion of *Ucp1* blunted LP-induced increases in energy expenditure but did not alter LP-induced FGF21 signaling on reducing body weight gain [72]. Most recently, a study demonstrated in a model of diabetic Lipodystrophy that 2 weeks of low-protein diet feeding increases FGF21 levels, reduces lipid droplets, and improves glucose uptake in BAT. In this same study, denervation of BAT blunted the benefits of a low-protein diet on thermogenetic effects to reduce body weight [73]. As reflected in Our bulk-RNA seq data, LP diet increased markers of brown adipose function on energy homeostasis such as bone morphogenetic protein 8B (*Bmp8b)* expression in BAT in WT mice. Other Genes that regulate oxidative stress, such as paraoxonase 1 (*Pon1*), an enzyme that hydrolyzes oxidized lipids and lipid hydroperoxides, were decreased by LP diet. Together, these data suggest that DPR significantly contributes to remodeling BAT and that FGF21 signaling is required to enhance thermogenic pathways.

## Limitations

In general, these studies show that DPR remodels adipose tissue to reduce markers of proto-oncogenes, senescence burden, and even preserve genes that regulate DNA repair during aging, yet it is important to comment on the limitations within our study. First, genetic variation can impact the metabolic benefits of diet on health or lifespan, and this variability in genetic response could extend to PR [74, 75]. Future studies will examine the effects of DPR in multiple strains or mixed background strains such as HET-3 or the Diversity Outbred [75, 76]. It is also important to consider how these findings of DPR are translatable to humans. New human data show that 5 weeks of protein restriction alters proteins in subcutaneous adipose tissue on improving mitochondrial function in lean young males [77]. In this same study, young mice were fed a low-protein/high-carbohydrate diet also showed enhanced mitochondrial function. And lastly, biological sex also alters the response to healthy diets [77]. The beneficial effects of PR have been primarily observed in males, and only males were used in the current study. Therefore, the extent of sexual dimorphism in the improvements in adipose tissue with DPR remains unclear. Finally, this study used only a single diet to model protein restriction (5% casein) and a single genetic model to delete FGF21 (*Fgf21* KO mice). A most recent study demonstrated that reducing the efficiency of the cysteine pathway requires FGF21 on enhanced browning in white fat independently of the canonical UCP1 signaling and improved measurable metabolic endpoints in both lean and obese mice [78]. It is also possible that the observed effects, including the contribution of FGF21, might vary with differing levels of protein content and uniquely interact with differences in sex or genetic background.

## Conclusion

A broad spectrum of diseases becomes more prevalent with aging, including metabolic syndrome (e.g., diabetes and atherosclerosis), neurodegenerative disorders, arthritis, increased cancer incidence, and heightened susceptibility to infections. Many of these conditions are associated with, exacerbated by, or driven by immunosenescence. To Our knowledge, this is the first investigation to characterize Gene expression patterns that underlie depot-specific responses of adipose tissue to prolonged dietary protein restriction and its interaction with fibroblast growth factor 21 (FGF21) signaling. In this study, we identify distinct metabolic and senescence-related gene expression profiles across three adipose tissue depots. Protein restriction was found to beneficially impact inguinal white adipose tissue (iWAT), epididymal white adipose tissue (eWAT), and brown adipose tissue (BAT) by enhancing metabolic processes (e.g., fatty acid metabolism, lipolysis in adipocytes, and insulin signaling) and reducing inflammatory pathways (e.g., viral response pathways, cytokine signaling, phagosome complement cascades, and blood complement activation), many of which were associated with FGF21-responsive gene networks. Collectively, these data provide a comprehensive blueprint of low protein-diet-induced molecular signatures—including FGF21-dependent mechanisms—that improve metabolic health, attenuate senescence-associated responses, and drive depot-specific genetic adaptations that may promote resilience and well-being during aging. These findings reveal novel FGF21-modulated targets of dietary protein restriction that could serve as promising therapeutic avenues for preserving adipose tissue function in aging.

## Supplementary Information

Below are the links to the electronic supplementary materials.

Supplementary tables (DOCX 22.8 KB)

Supplementary figures (PDF 1.37 KB)

Supplementary data (XLSX 7.78 KB)

## Data Availability

All data generated in this study are provided in the Supplementary Information/Source Data file and from the corresponding author upon reasonable request. Source data are provided with this paper.
