# Supplementary material for: Low protein-induced-FGF-21 signaling remodels adipose tissue on reduced markers of senescence during aging
Source: GeroScience. 2025 Sep 29;48(1):55–75. doi: 10.1007/s11357-025-01853-w (PMC12972293; doi:10.1007/s11357-025-01853-w)
Supplement: Supplementary file 1 — Supplementary tables (DOCX 22.8 KB) [file 11357_2025_1853_MOESM1_ESM.docx]

**Table S1. Composition of Diets**

| **Research Diets Cat No: Ingredient (g)** | **D11092301 5% Casein** | **D11051801 20% Casein** | **D11092308 HF-5%Casein** | **D11092309 HF-20%Casein** | | |
| --- | --- | --- | --- | --- | --- | --- |
| Casein | 50 | 200 | 50 | 200 | | |
| L-Cystine | 0.75 | 3 | 0.75 | 3 | | |
| Corn Starch | 485 | 375.7 | 134.1 | 0 | | |
| Maltodextrin 10 | 150 | 125 | 125 | 125 | | |
| Sucrose | 107.1 | 107.1 | 107.1 | 107.1 | | |
| Cellulose | 50 | 50 | 50 | 50 | | |
| Soybean Oil | 25 | 25 | 25 | 25 | | |
| Lard | 75 | 75 | 242 | 242 | | |
| Mineral Mix S10022C | 3.5 | 3.5 | 3.5 | 3.5 | | |
| Calcium Carbonate | 8.7 | 12.5 | 8.7 | 12.495 | | |
| Calcium Phosphate Dibasic | 5.3 | 0 | 5.3 | 0 | | |
| Potassium Citrate | 2.4773 | 2.5 | 2.4773 | 2.4773 | | |
| Potassium Phosphate | 6.86 | 6.86 | 6.86 | 6.86 | | |
| Sodium Chloride | 2.59 | 2.59 | 2.59 | 2.59 | | |
| Vitamin Mix V10037 | 10 | 10 | 10 | 10 | | |
| Choline Bitrartrate | 2.5 | 2.5 | 2.5 | 2.5 | | |
| FD&C Yellow Die #5 | 0 | 0.05 | 0 | 0.025 | | |
| FD&C Red Dye #40 | 0.05 | 0 | 0.025 | 0 | | |
| FD&C Blue Dye #1 | 0 | 0 | 0.025 | 0.025 | | |
| **Total** | 984.8 | 1001.3 | 775.9 | 792.6 | | |
|  |  |  |  |  | | |
|  | **D11092301** | **D11051801** | **D11092308** | **D11092309** | | |
| **Ingredient (g)** | **5% Casein** | **20% Casein** | **HF-5%Casein** | **HF-20%Casein** | | |
| **gm%** | | |  |  |  |  |
| Protein | 5 | 18 | 6 | 23 | | |
| Carbohydrate | 76 | 62 | 48 | 31 | | |
| Fat | 10 | 10 | 34 | 34 | | |
| **kcal%** |  |  |  |  | | |
| Protein | 4 | 18 | 4 | 18 | | |
| Carbohydrate | 74 | 60 | 37 | 24 | | |
| Fat | 22 | 22 | 59 | 59 | | |

**Table S2. List of Primers**

| Oligonucleotides |  |  |
| --- | --- | --- |
| *Il6 Forward* | *CTGGGAAATCGTGGAAT* | IDT |
| *Il6 Reverse* | *CCAGTTTGGTAGCATCCATC* | IDT |
| *Il1α Forward* | *GCAACGGGAAGATTCTGAAG* | IDT |
| *Il1α Reverse* | *TGACAAACTTCTGCCTGACG* | IDT |
| *Il1ß Forward* | *TCCTGTGTGATGAAAGACGGCAC* | IDT |
| *Il1ß Reverse* | *GTGCTGATGTACCAGTTGGGGAAC* | IDT |
| *Icam Forward* | *TTCACACTGAATGCCAGCTC* | IDT |
| *Icam Reverse* | *GTCTGCTGAGACCCCTCTTG* | IDT |
| *Mcp1 (ccl2) Forward* | *GCATCCACGTGTTGGCTCA* | IDT |
| *Mcp1 (ccl2) Reverse* | *CTCCAGCCTACTCATTGGGATCA* | IDT |
| *Timp1 Forward* | *GGGGTGTGCACAGTGTTTCC* | IDT |
| *Timp1 Reverse* | *ACCTGATCCGTCCACAAACA* | IDT |
| *mmp3 Forward* | *ACTCTACCACTCAGCCCAAGG* | IDT |
| *mmp3 Reverse* | *TCCAGAGAGTTAGACTTGGTGG* | IDT |
| *mmp12 Forward* | *CCTGCTTACCCCAAGCTGAT* | IDT |
| *mmp12 Reverse* | *ATGTTTTGGTGACACGACGG* | IDT |
| *Cd206 Forward* | *AAATGGCTTCCTGGAGAGCC* | IDT |
| *Cd206 Reverse* | *ACCCTCCGGTACTACAGCAT* | IDT |
| *Pnpla2 Forward* | *ACAGTGTCCCCATTCTCAGG* | IDT |
| *Pnpla2 Reverse* | *TTGGTTCAGTAGGCCATTCC* | IDT |
| *Cdkn1a (p21^Cip1^) Forward* | *GTCAGGCTGGTCTGCCTCCG* | IDT |
| *Cdkn1a (p21^Cip1^) Reverse* | *CGGTCCCGTGGACAGTGAGCAG* | IDT |
| *Cdkn2a (p16^ink4a^) Forward* | *CCCAACGCCCCGAACT* | IDT |
| *Cdkn2a (p16^ink4a^) Reverse* | *GCAGAAGAGCTGCTACGTGAA* | IDT |
| *Glb 1 Forward* | *CAAGACAGTGGCTGAAGCTCTG* | IDT |
| *Glb 1 Reverse* | *GAGGAAGCGTTGTTCGGTACAG* | IDT |

| **Critical Commercial Assays** | | **Source** |
| --- | --- | --- |
|  |  |  |
| FGF21 ELISA | Biovendor | RD291108200R |
| Adiponectin ELISA | EMD Millipore | EZMADP-60K |
|  |  |  |
| **Experimental Models: Organisms/Strains** |  |  |
| Mouse:FGF21-KO | Dr. Steven Kliewer | Potthoff MJ, Inagaki T, Satapati S, et al. FGF21 induces PGC-1alpha and regulates carbohydrate and fatty acid metabolism during the adaptive starvation response. *Proc Natl Acad Sci U S A*. 2009;106(26):10853-10858. doi:10.1073/pnas.0904187106 |
| Mouse: Aged C57BL/6J Mice | The Jackson Laboratory | stock number 000664 |

**Table S3. OTHER REAGENT or RESOURCE**
