# Supplementary material for: Low protein-induced-FGF-21 signaling remodels adipose tissue on reduced markers of senescence during aging
Source: GeroScience. 2025 Sep 29;48(1):55–75. doi: 10.1007/s11357-025-01853-w (PMC12972293; doi:10.1007/s11357-025-01853-w)
Supplement: Supplementary file 2 — Supplementary figures (PDF 1.37 KB) [file 11357_2025_1853_MOESM2_ESM.pdf]

Supplemental Figure 1

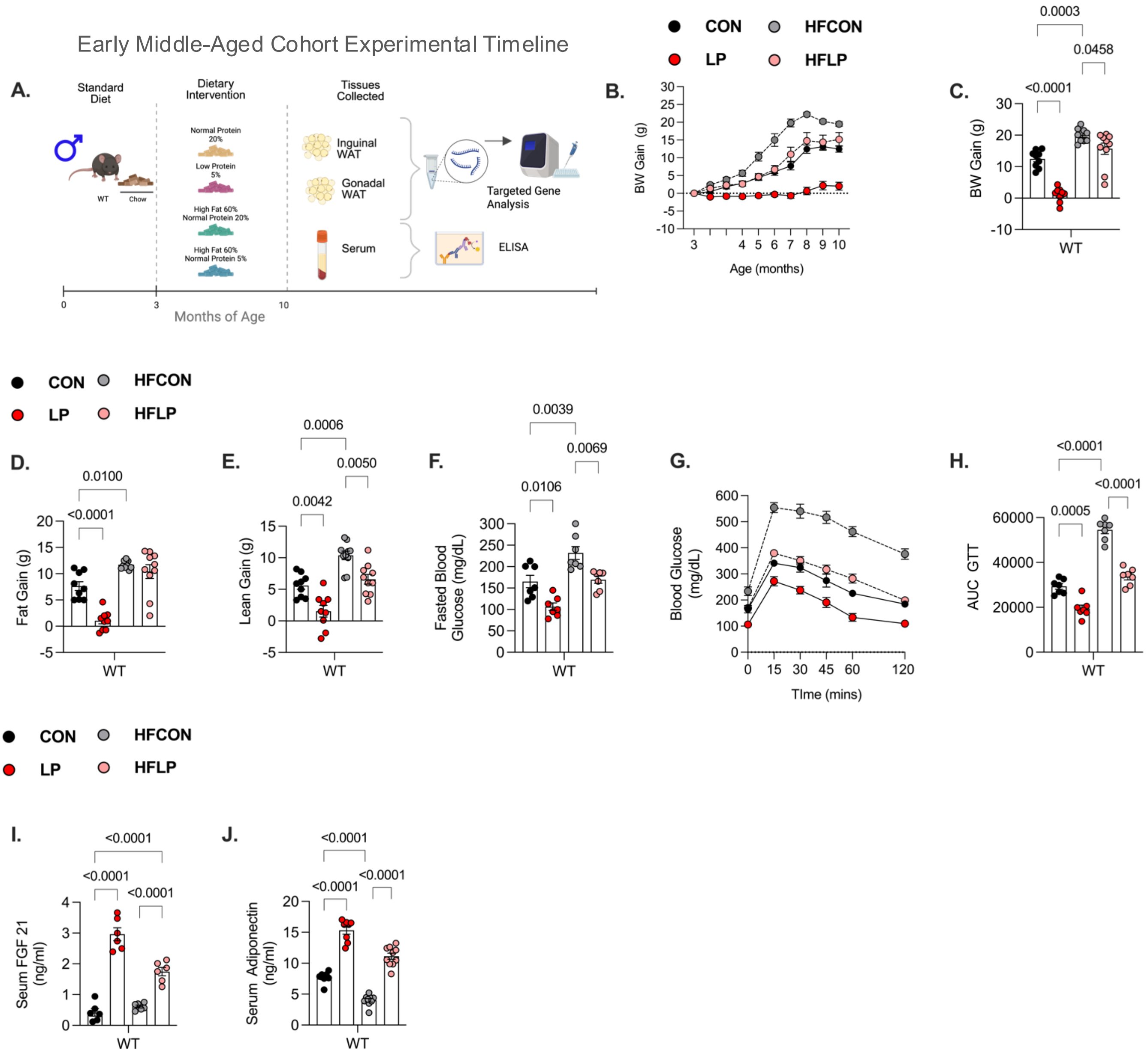

Supplemental Figure 1. Low-protein diet improves metabolic endpoints at early middle age and during obesity**. A.** Graphical methodology: C57BL/6 male mice were placed on CON, LP, HFCON, HFLP at 3 months of age (8-10 mice /group), various metabolic endpoints on BW and glucose homeostasis throughout the feeding phase of the study as indicated, and tissue collection at 10 months of age. **B** Body weight gain over time from initiation of diet (n = 10 mice/group). **C** Terminal Body weight gain. **D** Fat gain. **E** Lean gain. **F** Fasting blood glucose at 9 months of age. **G** Glucose tolerance test conducted at 9 months of age (n=7 mice/diet). **H** Area under the curve glucose for the GTT. **I** Serum FGF21 levels. **J** Serum adiponectin levels. Statistical analyses were conducted using one-way ANOVA. All values are mean ± SEM, with significant main effects of protein or *post hoc* comparison within the fat*protein interaction.

Supplemental Figure 2

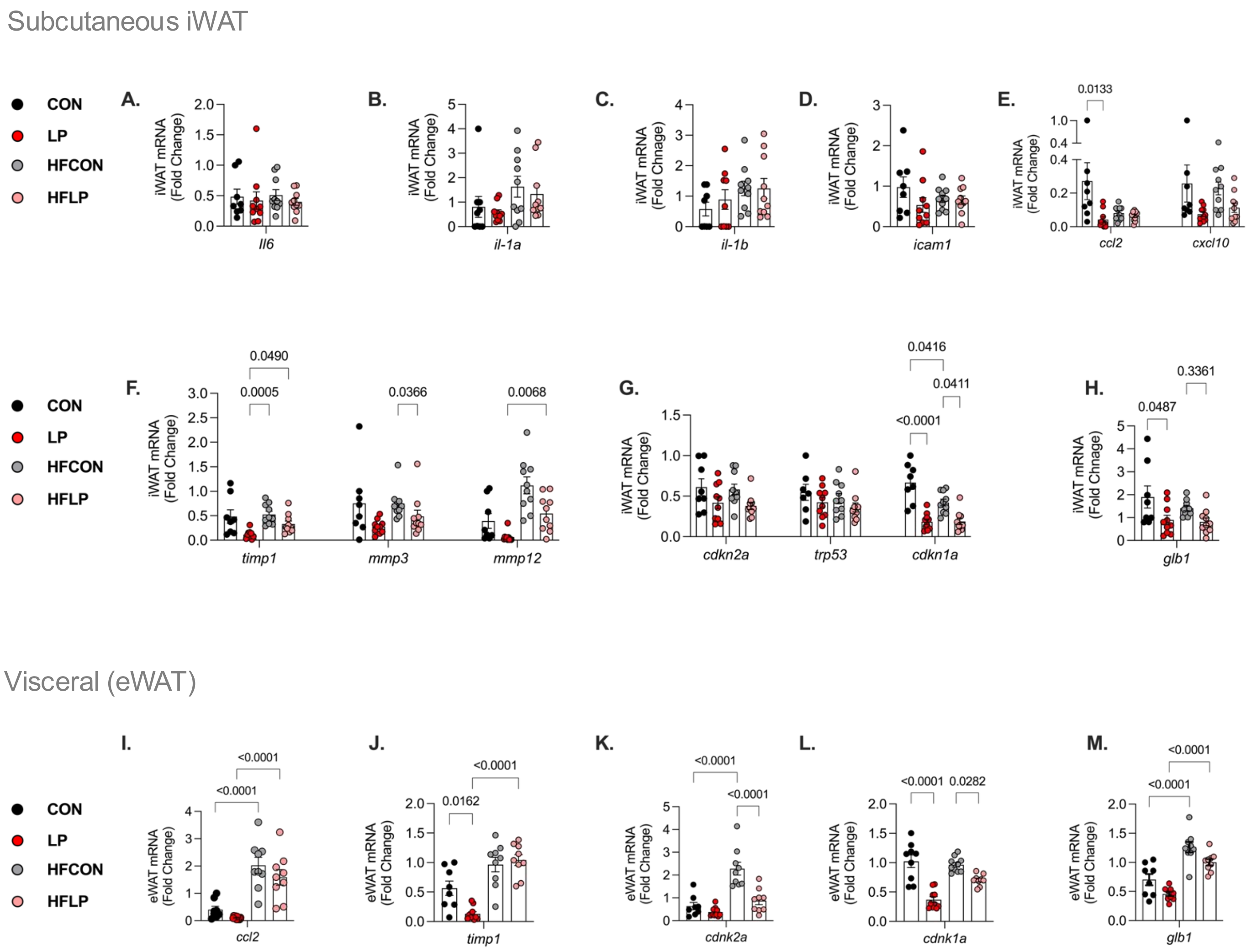

Supplemental Figure 2. Low-protein diet reduces senescence-related markers in inguinal white adipose tissue at early-middle age and during obesity**. A-H.** Subcutaneous iWAT from C57BL6 (WT); Fold change of SASP-related markers, interleukins (IL), monocyte and chemoattract mRNA expression in iWAT at 10 months of age, *n*= 8(CON), 10 (LP), 10 (HFCON), 10(HFLP)**. A** Mouse interleukin -*Il6.* **B** *Il1a.* **C** *Il1b.* ***D*** Leukocyte adhesion molecule *Icam1.* **E** *Ccl2 and Cxcl10.* **F** Matrix metalloproteinases *Timp1, Mmp3 and Mmp12.* **G** Markers of cell cycle arrest *Cdkn2a,* tumor protein *p53,* and *Cdkn1a* **. H** lysosomal beta-D-galactosidase *Glb1.* **I-M** Fold change of SASP-related markers of cellular arrest, cell proliferation, and monocyte chemoattract mRNA expression in visceral (eWAT) adipose tissue of WT mice at 10 months of age, *n*= 8(CON), 10 (LP), 10 (HFCON), 10(HFLP). **I** *Ccl2.* **K** *Timp1.* **L** *Cdkn1a.* **J** *Cdkn2a.* **M** beta-D-galactosidase *Glb1* Statistical analyses were conducted using one-way ANOVA. All values are mean ± SEM, with significant main effects of protein or *post hoc* comparison within the fat*protein interaction.

Supplemental Figure 3.

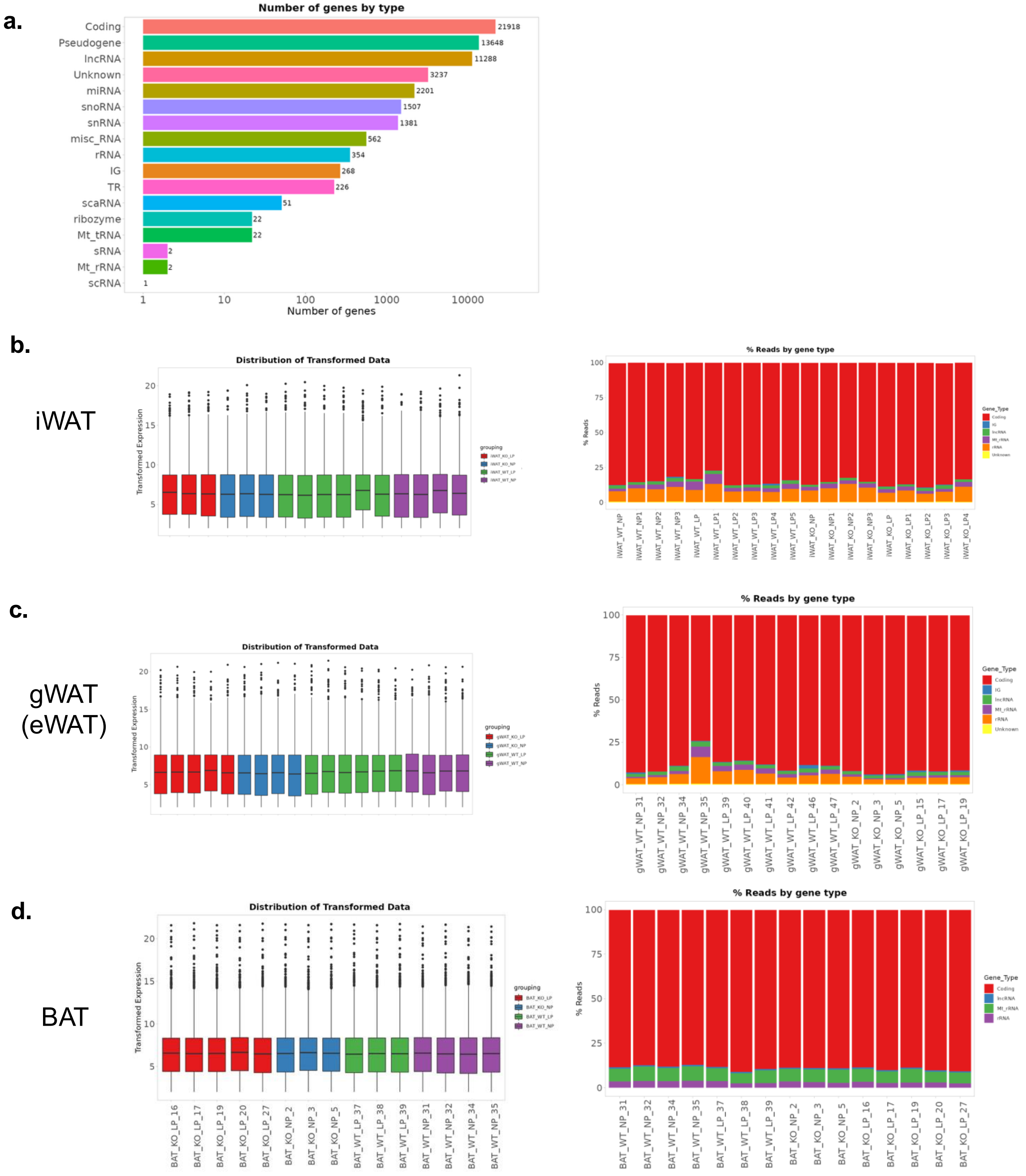

Supplemental Figure 3. Bulk RNA-seq Transformative data of adipose depots that was used to prepare the data for differential expression statistical analysis. Briefly, a total of 21918 coding genes were identified in iWAT (19 samples), eWAT (16 samples) and BAT (15 samples) and used to examine *genotype* x *diet* effects in the following comparisons: WT-LP vs *Fgf21* KO-LP and WT-CON vs *Fgf21* KO-CON for genotype by diet interaction; and WT-CON vs WT-LP; *Fgf21* KO-CON vs *Fgf21* KO-LP for diet interaction within same genotype. DESeq2 analysis parameters were defined to screen DEGs at a $|\log_2$ fold change$| > 1$ and a FDR $<$ 0.05. Next, DEGS were used for parametric analysis of Gene Set Enrichment Analysis (PGSEA) to perform KEGG Pathway analysis (Kyoto Encyclopedia of Genes and Genomes) and GO: Biological Processes (GO: The Gene Ontology).

Supplemental Figure 4.1

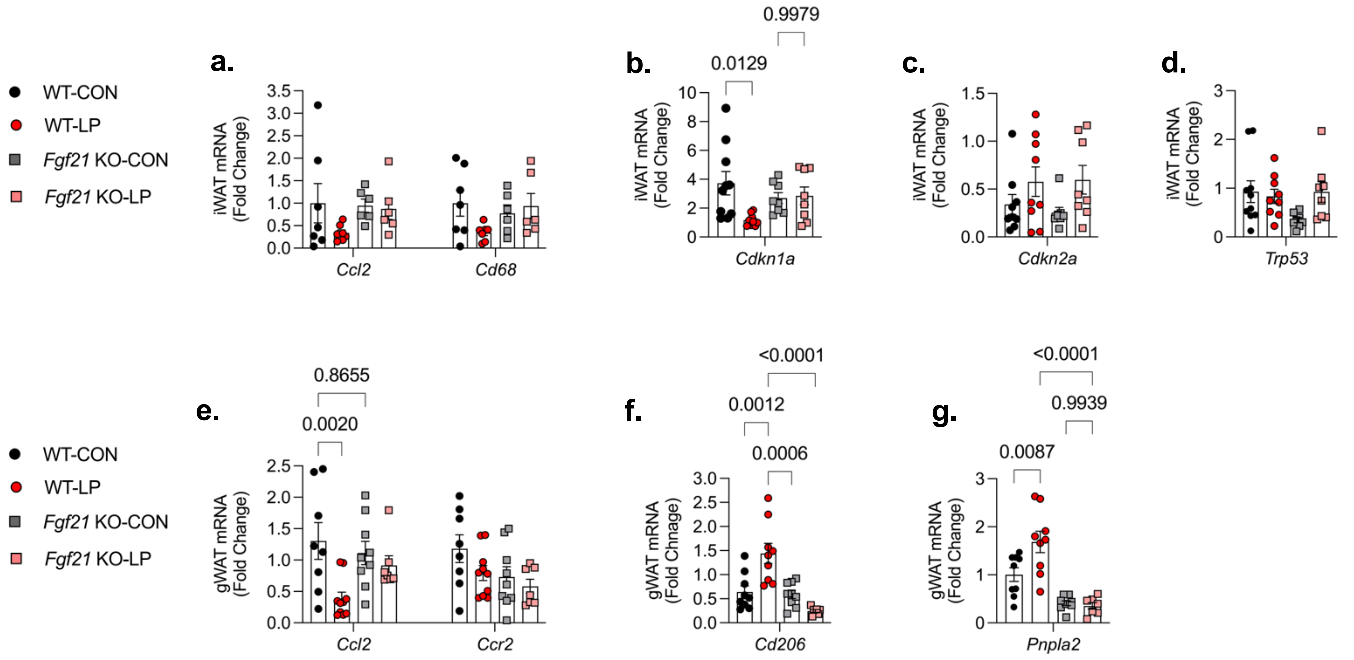

Supplemental Figure 4.1 . FGF-21 is dependent and independent on remodeling adipose tissue on inflammatory, anti-inflammatory and senescence burden at 22 months of age. **A** Graphical methodology: subcutaneous (iWAT), visceral (gWAT),and brown (BAT) adipose tissue from a previous study of C57BL/6 (WT) and *Fgf*21 KO mice fed either normal-protein (CON) or low-protein (LP) at 3 months of age until 22 months of age. **B-D** Fold change in mRNA expression in iWAT. **B** Monocyte marker *Ccl2* and *Cd68.* **C** *Cdkn1a.* **D** *Cdkn2a***.** **E** Tumor protein *p53.* **E-G** Fold change in mRNA expression in eWAT. **E** Monocyte maker *Ccl2* and *Ccr2.* **F** M2 macrophage marker *Cd206.* ***G*** Triglyceride hydrolase *Pnpla2*. Statistical analyses were conducted using two-way ANOVA. All values are mean ± SEM, with significant main effects of diet or *post hoc* comparison within the diet*genotype interaction.

Supplemental Figure 4.2

a. Inguinal WAT

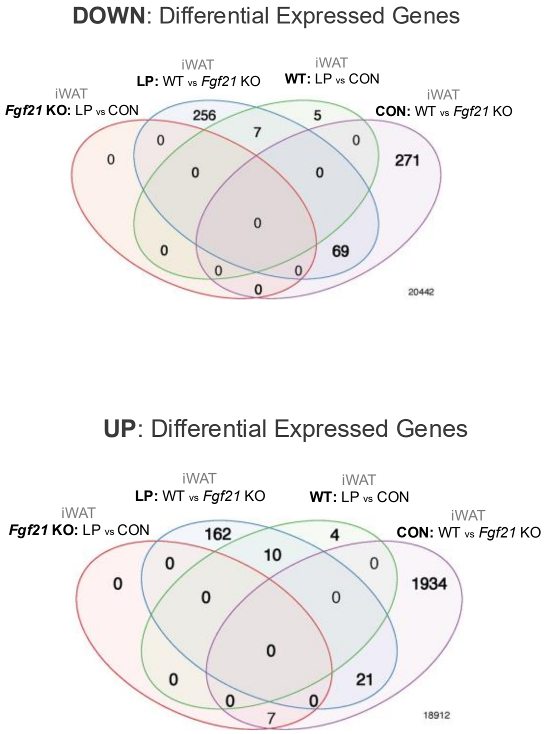

c.

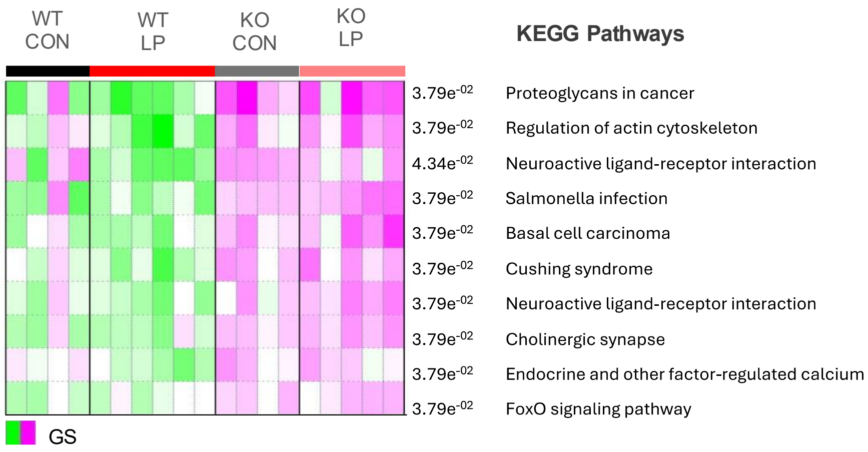

Supplemental Figure 4.2. **a.** Low-Protein Diet Induces FGF21-dependent Transcriptional Signatures On Improved Neurovascular Remodeling in Brown Adipose Tissue**. iWAT** from a previous study was used to preform bulk RNA-seq from C57BL/6 (WT) and *Fgf21* KO mice fed either normal protein control (CON) and low-protein (LP) diet at 3 months of age until 22 months of age. Venn diagrams of downregulated (left panel) and upregulated (right panel) DEGS followed by PGSEA: Parametric Gene Set Enrichment Analysis for KEGG pathways

b. Epididymal WAT

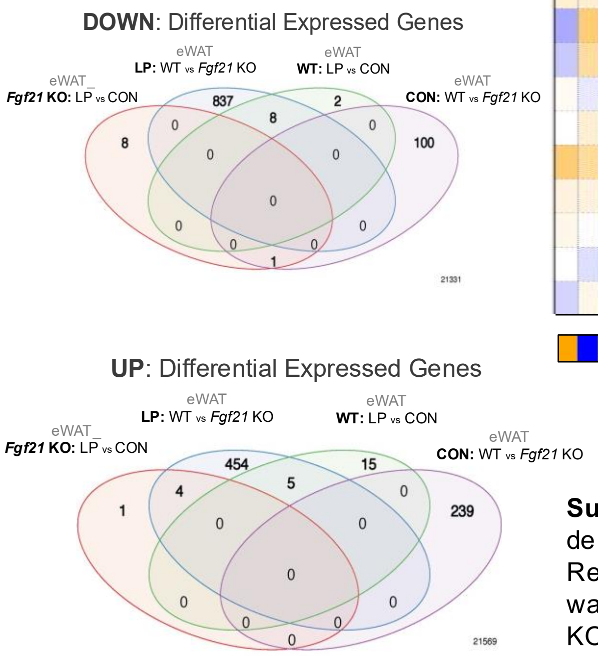

d.

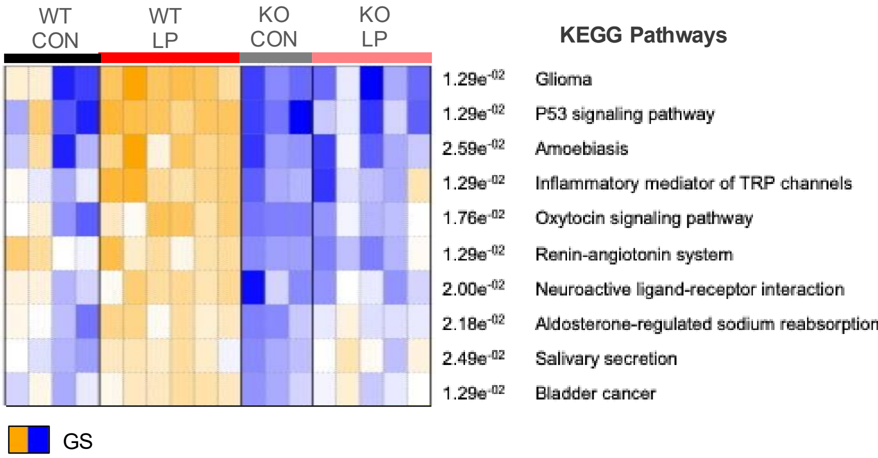

Supplemental Figure 4.2. **b.** Low-Protein Diet Induces FGF21-dependent Transcriptional Signatures On Improved Neurovascular Remodeling in Brown Adipose Tissue**. eWAT** from a previous study was used to preform bulk RNA-seq from C57BL/6 (WT) and *Fgf21* KO mice fed either normal protein control (CON) and low-protein (LP) diet at 3 months of age until 22 months of age. Venn diagrams of downregulated (left panel) and upregulated (right panel) DEGS followed by PGSEA: Parametric Gene Set Enrichment Analysis for KEGG pathways

Supplemental Figure 5

a. BAT

DOWN: Differential Expressed Genes

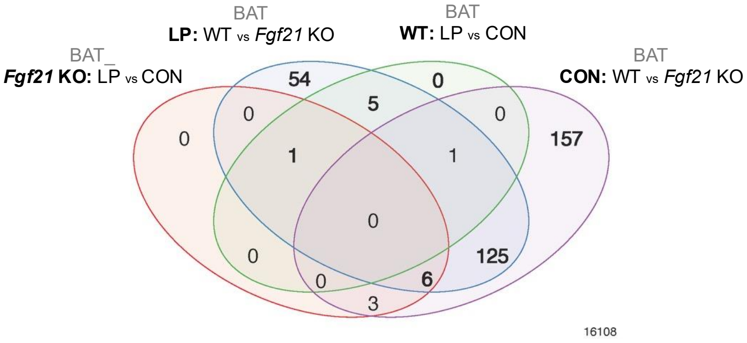

UP: Differential Expressed Genes

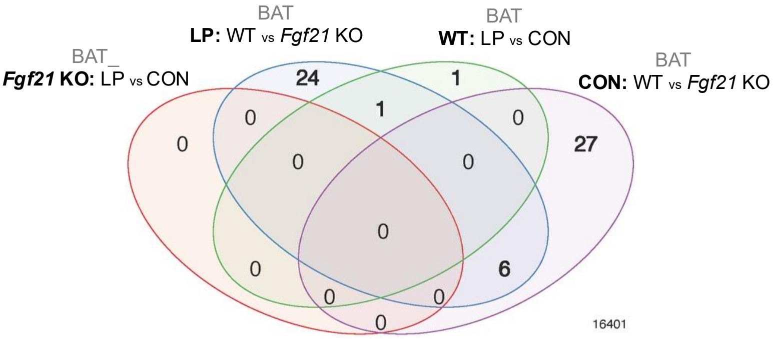

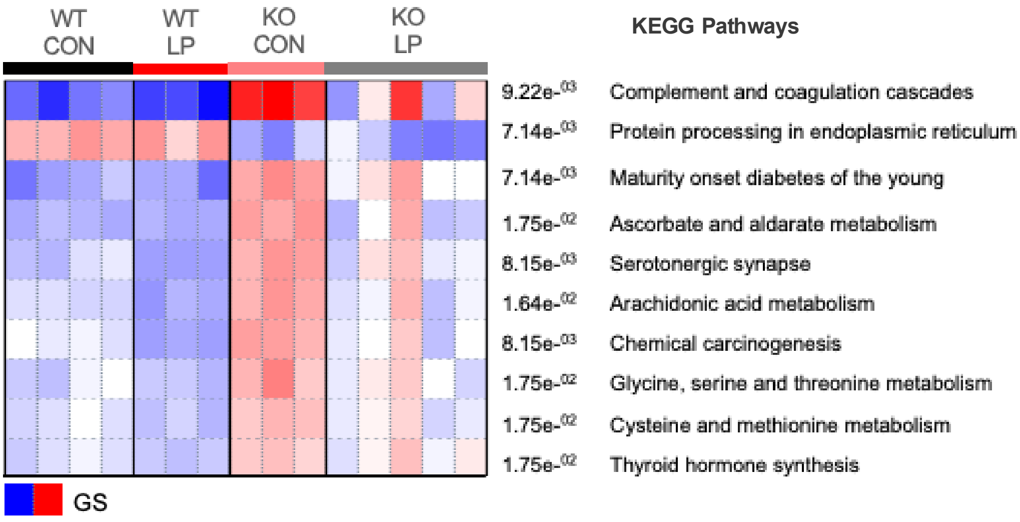

Supplemental Figure 5. **a.** Low-Protein Diet Induces FGF21-dependent Transcriptional Signatures On Improved Neurovascular Remodeling in Brown Adipose Tissue**. BAT** from a previous study was used to preform bulk RNA-seq from C57BL/6 (WT) and *Fgf21* KO mice fed either normal protein control (CON) and low-protein (LP) diet at 3 months of age until 22 months of age. Venn diagrams of downregulated (left panel) and upregulated (right panel) DEGS followed by PGSEA: Parametric Gene Set Enrichment Analysis for KEGG pathways
